# Unveiling Carbonic Anhydrase VIII, X, XI Expression in Cancer and Neurological Diseases Through Integrated Bioinformatics Approaches

**DOI:** 10.1177/11779322261475822

**Published:** 2026-08-03

**Authors:** Ratul Bhowmik, Rajarshi Ray, Ashok Aspatwar

**Affiliations:** 1 Faculty of Medicine and Health Technology, 162225 Tampere University, Tampere, Finland

**Keywords:** carbonic anhydrases, carbonic anhydrases related proteins, cancer, neurological diseases, transcription factors, biomarkers, biological networks, RASAR

## Abstract

Carbonic anhydrases are metalloenzymes found both in vertebrates and invertebrates. The CAs catalyze the reversible hydration of CO_2_ to bicarbonate and H+ ions and play a significant role in respiration, transport of CO_2_, pH homeostasis, electrolyte secretion, and biosynthetic reactions. The enzymatic activity of CAs is due to the coordination of Zn^2+^ in the active site by three histidine residues; however, in humans, there are three CAs known as CA-related proteins (CARPs) that are catalytically inactive due to the absence of one or more of the three histidine residues required for the coordination of the Zn^2+^ in the active site. Studies have shown that CARPs are expressed in all parts of the brain and are overexpressed in some cancers suggesting that the CARPs play a crucial role in neurological disorders and the development of cancer. However, the precise physiological roles of CARPs are still an enigma. In this study, we present a comprehensive biological workflow that employs various machine learning methodologies and statistical procedures to assess the similarity across CARPs by evaluating shared biological parameters. This approach enabled us to identify potential biomarkers, including transcription factors, co-expressed genes, and phenotypes, that may influence the expression of CARPs within disease pathways. Furthermore, we proposed a computational human health model by analyzing drugs and chemical candidates to prioritize compounds that may modulate regulatory networks associated with CARPs. These computational analyses identified candidate compounds for future experimental investigation in the context of neurological disorders and cancer.

## Graphical abstract

**Graphical Abstract:**
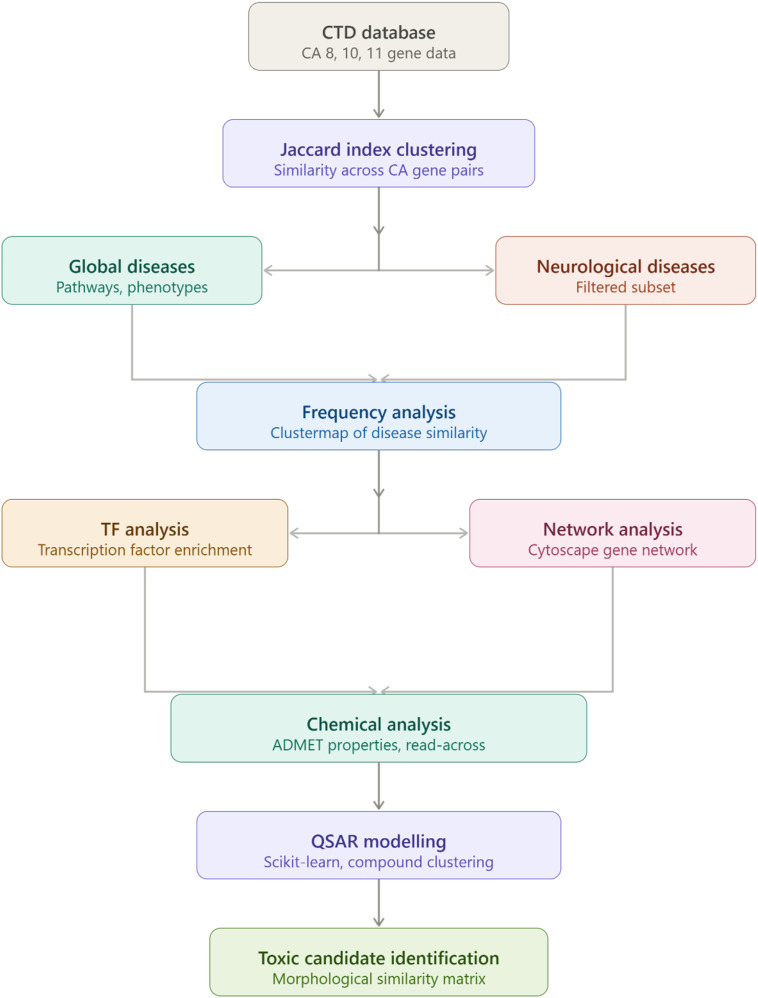

## 1. Introduction

Carbonic anhydrases (CAs, EC 4.2.1.1) are ubiquitous metalloenzymes found in vertebrates and invertebrates, mainly containing zinc as a metal ion.^1^ The fundamental role of CAs is the reversible hydration of carbon dioxide to bicarbonate and hydrogen ions in a reaction CO_2_ + H_2_O ⇋ HCO_3_^−^ + H^+ 1^. The biological significance of CAs mainly lies in their involvement in various biological processes, namely photosynthetic reactions, acidification within renal tubules, respiratory pathways, and bone resorption.^1-6^ The CAs belong to evolutionarily eight different gene families and are identified by Greek letters: α, β, γ, δ, ζ, η, θ, and ι.^1^ The animal kingdom contains only CAs belonging to the -α class, and humans are known to contain 16 α-CAs.^1^ Among the α-CA, thirteen CAs, namely, CA I, II, III, IV, VA, VB, VI, VII, IX, XII, XIII, XIV, and XV, are catalytically active. In the catalytically active CAs, zinc in the active site is coordinated by three histidine residues essential for the enzyme’s catalytic activity. The catalytic activity of the CAs is necessary for maintaining the overall biological reactions associated with the modulation of various biological pathways involved in the healthy functioning of the tissues and organs.^1,3,7,8^ The remaining three CAs are catalytically inactive and are known as carbonic anhydrase-related proteins (CARPs).^2,4,9-12^ The catalytic inactivity of the CARPs is due to the absence of one or more of the three histidine residues required for the coordination of the zinc atom in the activity site.^4,9,13^ Studies related to CARPs mainly include gene expression studies conducted using western blot, RT-PCR techniques, and immunohistochemistry within the brain tissues of humans, mice, and zebrafish.^9,14-16^ These studies have shown that CARPs are predominantly expressed in the brain and central nervous system. Among the CARPs, CARP VIII is predominantly expressed in the cerebellar region of the brain and, more specifically, in the Purkinje cells, whereas CARP X and CARP XI are expressed in all parts of the brain.^1,2,4,9,15,16^ The expression studies on the CARPs showed that these proteins are localized along the brain tissues, denoting the differential expression of CA VIII, X, and XI within neural tissues, and hence could be potentially connected to the modulation of proteins through interaction with other proteins associated with the development of brain tissues and contribute to neural functioning, hence leading to the proper functioning of neurological health among humans.^17-20^ Studies have shown that the mutations in the *CA8* gene resulted in neurological disorders like ataxia and gait disorders in mice.^21^ The spontaneously occurring mutation in the *CA8* gene in the members of Saudi Arabian and Iraqi led to mental retardation and ataxia and quadrupedal gait, respectively.^22,23^ MRI analyses of affected family members revealed reduced cerebellar volume, suggesting that mutations in the *CA8* gene contribute to cerebellar abnormalities that impair motor coordination and cognitive development.^22^ In addition, knockout and knockdown studies in zebrafish showed that these proteins are crucial for brain development, and defects in these proteins lead to ataxia and motor coordination defects.^1,14-16^

Similarly, studies conducted on cancer cell lines and malignant tissues provided the inference that CARP VIII, CARP X, and CARP XI were more involved in the cancer development pathways, which were mainly found to localize within gastrointestinal tracts and pulmonary tissues.^2,4,24,25^ Moreover, CA VIII has also been found to be involved in various carcinogenic conditions, which are mostly localized within fetal cells during their development and also found within colon and pulmonary tissues, leading to cancerous development within them.^24^ Similarly, research conducted on CA X and XI also produced similar results, pointing to their potential connections among cancer development pathways and involvement within the neurological functioning of human beings, which, during events of malfunction, led to the development of disorders that affected the neurological tissues and also led to cancer along multiple organs.^26,27^

Recent CA drug-discovery studies further demonstrate the therapeutic relevance of targeting CA isoforms in cancer and neurological disease-associated contexts. Sulfonamide and triazole-based hCA inhibitors have been designed against several human CA isoforms, including the tumor-associated hCA IX and hCA XII, with studies integrating enzyme inhibition, anticancer cytotoxicity, and molecular docking analyses. Similar structure-guided strategies have also been applied to naphthoquinone–thiazole hybrids, thiosemicarbazone derivatives, and dual hCA/acetylcholinesterase inhibitors, highlighting the relevance of CA modulation in neurodegenerative, glaucoma-related, and cancer-associated pharmacological frameworks. In addition, tail-approach-based inhibitor design has been used to improve isoform selectivity across hCA I, II, IX, and XII. These recent advances provide a strong rationale for integrating CA/CARP-associated disease signatures with transcriptional, chemical-space, ADME, and machine-learning-based prioritization strategies.^28-30^

Additionally, certain prior research has used gene expression analyses to examine the expression patterns of members of the carbonic anhydrase family in various tissues. For instance, Çankaya et al (2007)^31^ examined the expression of genes that code for catalytic proteins, such as a number of carbonic anhydrase isoenzymes, offering important information about their tissue-specific expression profiles. By concentrating on the catalytically inactive carbonic anhydrase-related proteins (CA VIII, CA X, and CA XI) and combining multi-omics, regulatory networks, pathway enrichment, and machine learning techniques to investigate their possible roles in cancer and neurological disorders, our study expands on these findings.

Based on the procedures and research studies mentioned, we conducted a comprehensive analysis using various clustering machine learning algorithms within a computational workflow. This analysis included the identification of Transcription Factors (TFs) which are likely associated with the biological connections related to Carbonic Anhydrase-Related Proteins (CARPs) and their role in the development of neurological diseases and cancer pathways. Our ultimate goal was to create a computational human health model capable of analysing candidate drugs and chemical candidates with possible potential therapeutic effects against disease targets related to these carbonic anhydrases. This was achieved by developing a read-across quantitative structure-activity relationship (RASAR) model and thoroughly examining the overall biological activity of drugs against CA VIII, X, and XI.

## 2. Materials and Methods

To establish the biological connections between CA VIII, CA X, and CA XI, in their involvement in the development of cancer and neurological disorders, we employed an end-to-end approach. This approach utilized similarity matrices, such as the Jaccard Index (JI) and hierarchical clustering (HC), to identify shared biological parameters, including pathways and symptomatic phenotypes, among these CAs. By analyzing these similarities, we were able to uncover common developmental mechanisms underlying both cancer and neurological disorders associated with these carbonic anhydrases. Since CA VIII, CA X, and CA XI, are catalytically inactive carbonic anhydrase-related proteins (CARPs), the present study aimed to identify transcription factors (TFs) and co-expressed genes that are **computationally associated** with their expression. These analyses were performed to prioritize regulatory networks, biological pathways, human phenotypes, tissue- and cell line-specific expression patterns, and disease associations potentially linked to neurological disorders and cancer. This comprehensive computational workflow enabled the identification of candidate biomarkers and regulatory relationships for further experimental investigation. Additionally, to identify chemical candidates that could potentially modulate pathways associated with neurological and cancer-related conditions, we analyzed drugs associated with each of the TFs linked to the CARPs, focusing on their structural and pharmacokinetic properties, which may provide insights into their potential biological relevance and suitability as computationally prioritized candidate compounds for future experimental evaluation. Additionally, we included a set of non-drug chemical candidates known from drug databases to contribute to toxic responses in patients with cancer and neurological disorders. We then employed a machine learning (ML) based clustering algorithm to develop an automated model that groups chemical candidates based on their similarity in structural descriptors and various pharmacokinetics properties, clustering these candidates together based on the degree of similarity in their shared values. In the following sections, we detail the methodologies and resources used to create an end-to-end analytical workflow for assessing the biological connections associated with CAs in cancer development and neurological disorders. A summary of the computational workflow, analytical tools, databases, inputs, and outputs used throughout the study is presented in Table 1.

**Table 1.** Summary of the Computational Workflow, Databases, Software Tools, Analytical Phases, Inputs, and Outputs Used in This Study

Phase	Tool/Database	Purpose	Input	Output
1 — Data curation	CTD (ctdbase.org)	Disease, pathway, phenotype and chemical data for CAs	CA 8, CA 10, CA 11 gene identifiers	Raw dataset: diseases, pathways, phenotypes, chemical candidates
1 — Data curation	Python — Pandas, NumPy	Pre-processing, deduplication, partitioning	Raw CTD dataset	Cleaned, partitioned dataset: neurological vs global disease classes
2 — Clustering	Python — Seaborn, Matplotlib	Jaccard index similarity matrix + hierarchical clustering heatmaps	Partitioned disease/pathway/phenotype datasets	JI clustermap heatmaps with dendrograms per CA pair
2 — Clustering	wordcloud (Python, pypi.org)	Frequency visualisation of enriched diseases, pathways, phenotypes	Cluster outputs from JI heatmaps	Word cloud frequency maps for neurological and global disease classes
3 — TF analysis	X2K/Enrichr (maayanlab.cloud)	Transcription factor identification; pathway and phenotype enrichment	*CA8, CA10, CA11* gene identifiers	Ranked TF list; enriched biological pathways, phenotypes, disease sets
3 — TF analysis	Cytoscape (cytoscape.org)	Co-expression gene network construction and visualisation	TF-associated co-expressed genes for each CA	Gene interaction network; tissue and cancer cell-line expression sites
3 — TF analysis	GSEA/MSigDB (gsea-msigdb.org)	Cancer gene expression heatmaps (microarray and RNA-seq)	Co-expressed gene sets from TF analysis	Up/downregulation heatmaps across cancer-related biological pathways
4 — Chemical analysis	PubChem/DrugBank	Classification of candidates as therapeutic (drug) or toxic (non-drug)	Chemical candidates linked to TFs	Two separate lists: therapeutic and toxic chemical candidates
4 — Chemical analysis	PaDEL Descriptor (yapcwsoft.com)	Molecular fingerprint and substructure descriptor calculation	SMILES/structures of therapeutic and toxic candidates	Substructure and PubChem-based molecular fingerprint matrices
4 — Chemical analysis	SwissADME (swissadme.ch)	ADMET pharmacokinetic property calculation	Chemical structures of candidate compounds	ADMET parameter profiles (absorption, distribution, metabolism, excretion, toxicity)
4 — Chemical analysis	Python — Seaborn	Pearson correlation similarity matrix heatmap	Fingerprint + ADMET descriptor matrices	Compound similarity heatmaps for therapeutic and toxic lists
5 — RASAR/ML	scikit-learn (scikit-learn.org)	KMeans clustering, KNN classifier, PCA, grid-search optimisation	PCA-transformed fingerprint + ADMET dataset (80/20 train/test split)	Cluster assignments; trained KNN model; accuracy scores, confusion matrices, PCA scatter plots

### 2.1. Curation and Pre-Processing of Biological Data

We obtained the dataset related to the diseases, biological pathways, and phenotypes associated with CA VIII, CA X, and CA XI,, respectively, from the Comparative Toxicogenomics Database (CTD) [https://ctdbase.org/].The raw dataset consisted of information for each of the CA categories along with its respective biological conditions and diseases associated with several neurological disorder types Additionally potential chemical candidates and molecular components associated with the treatment or other possible biochemical impact were also present for each of the CA data. . These disease types included conditions, which encompassed specific diseases localized within the CNS and spinal cord, and global diseases, which encompassed diseases localized in organs and tissues other than the brain. Following this, we removed the redundant and null values from the dataset which consisted of empty data related to CAs and duplicate data containing same values for CA types, causal disease/condition and chemical factors respectively and arranged the dataset, considering attributes like diseases, , and chemical components, respectively, with each class of CAs. Additionally we downloaded two other datasets from CTD respectively which consisted of the biological associations between diseases and phenotypes and another having connections across various chemical candidates along with their specific directional phenotypic effects within the human anatomy. These datasets are utilised in our study to develop possible connections across the CARPs and understanding their impact in the progression of neurological disorders. This analysis highlighted their potential mechanisms of action in the progression of diseases, whether neurological or cancerous. Ultimately, the annotated dataset was utilized to develop similarity cluster maps and to perform TF determination analysis, as outlined in the subsequent steps.

### 2.2. Machine Learning-Assisted Clustering Analysis for Understanding Shared Biological Attributes of CA VIII, CA X, and CA XI

We utilized the dataset obtained from the mentioned process to create a clustering architecture to understand the significance of complex biological networks. This architecture employed the Jaccard Index (JI) and distance-based hierarchical clustering (HC) techniques in Python programming language [https://www.python.org/]. It allowed us to identify the common biological pathways, phenotypes, and disease categories shared among the three CAs. The similarity scores assigned to each CA were based on the rate of similarity between each pair of CAs. In addition, by utilizing specific Python-based libraries such as Pandas [https://pandas.pydata.org/], Numpy [https://numpy.org/], Matplotlib [https://matplotlib.org/], and Seaborn [https://seaborn.pydata.org/], we successfully converted the similarity-based matrix into a more easily understandable graphical format. This format displayed clusters of similar CAs represented by similar colors, with distance-based dendrograms connecting the genes. The distance of the branched dendrogram and the color of the cluster indicated the similarity between each pair of CAs. By performing this analysis, we were able to determine the various neurological diseases and cancer conditions associated with CA types and understand which of the considered CAs were majorly involved in the development of those diseases. In addition, we gained insight into the structure of several biological pathways and characteristics related to the advancement of CA in humans by analyzing the JI map created using pairings of genes connected with these traits. The clustering analysis enabled the identification of similarities among CA VIII, CA X, and CA XI based on their shared biological attributes, thereby facilitating the prioritization of disease associations and biological pathways for subsequent analyses. To thoroughly evaluate the clustering outcome, we additionally generated frequency word clouds. These word clouds, which will be discussed in the following sections, offer specific information about the diseases, biological pathways, and phenotypes that were identified within the clusters formed by the CA pairs. To calculate the similarities between the pairs, the Jaccard Index (JI) first applies set theory to identify the common elements between each pair. This number is then divided by the total number of elements across both samples in the pair, resulting in a standardized score, where a higher score indicates greater similarity between the samples. The Hierarchical Clustering (HC) methodology further employs distance calculation by comparing the JI scores using subtraction. In this context, a lower distance indicates that the data points are closer to each other, suggesting greater similarity between the samples due to their minimal differences. Thus, by using these procedures, we can visually identify biological attributes like diseases, pathways, and phenotypes that are biologically clustered with one another. This enables the identification of samples with similar biological properties that can be prioritized for further computational and experimental investigation. The following lists the equations that the mentioned mathematical techniques use. The JI formulation that operates on the mentioned pairs of samples, denoted by A and B, respectively, is visible from equation (i). However, the formulation employed by the distance-based HC, which is defined as the Euclidean distance in this case, is equation (ii), which is used to calculate the differences among each data point based on their frequency and occurrences. As a result, low difference values indicate that the samples are similar, and vice versa.

1 $$JI\left(A,B\right)=\frac{A\cap B}{A\cup B\;}$$

2 $$\sqrt{\overset{n}{\underset{i=1}{\sum }}\left(xi-yi\right)2}$$

### 2.3. Pathway, Phenotype, and Gene Ontology Analysis

After analyzing the JI and HC heatmaps across pairs of CA VIII, CA X, and CA XI1 concerning the various disease categories of neurological and global diseases, respectively, along with their subsequent biological pathways and phenotypes, we utilized the wordcloud library [https://pypi.org/project/wordcloud/] from Python to construct a graphical representation pointing out the specific diseases, biological pathways and phenotypes across the clusters from the JI maps, which are occurring more frequently. As mentioned previously, we categorized our CA dataset into two classes: the neurological disease class relating to diseases localizing along the neural and spinal tissues, respectively, and the global disease class, which consisted of diseases across all other organs except the brain, which were found to be mostly related to cancer and carcinogenic conditions, respectively. Considering the JI maps for each disease distribution across CA pairs, we deployed the wordcloud approach to obtain the respective frequency maps for neurological disease and global diseases, respectively, to determine which specific disease conditions appear more significantly than others, denoting their biological occurrences, respectively. Additionally, we also assessed the frequency counts for the biological pathways and phenotypes that were enriched within each disease category, respectively, and their involvement across the expression of the mentioned CAs, respectively. Therefore, by implementing this particular step in our approach, we effectively identified the diseases, pathways, and characteristics that could serve as potential biomarkers for detecting cancer and neurological abnormalities concerning CAs. These biomarkers have been found to be closely associated with the expression of cancer-related genes and can assist in developing drugs that target the transcription factors associated with disease progression. By engaging specific biological pathways, this targeted approach can ultimately influence the expression of characteristics associated with these diseases.

### 2.4. Determination of Co-Expressed Genes and Transcription Factors Concerning Cancer and Neurological Disorders

In the previous section of our manuscript, we mentioned that CA VIII, CA X, and CA XI, are part of the carbonic anhydrase family but are considered inactive enzymes. This inactivity is due to minor structural changes that affect the arrangement of specific amino acids, which in turn impacts the enzyme’s reactivity and overall biological function. As a result, these enzymes cannot directly contribute to the development of various neurological and cancer-related phenotypes by modulating the biological pathways that lead to disease progression. . Another potential factor could be the involvement of various transcription factors (TFs), which are proteins that regulate the transcription and translation of DNA and mRNA associated with the formation of the mentioned CAs. These TFs may regulate the expression of CA VIII, CA X, and CA XI, and their associated gene networks through transcriptional regulatory mechanisms. Therefore, we investigated TFs that are computationally associated with CARP expression to identify candidate regulatory pathways and biomarkers linked to neurological disorders and cancer. . Hence, in our manuscript, using the X2K and Enrichr server [https://maayanlab.cloud/X2K/] [https://maayanlab.cloud/Enrichr/], we identified transcription factors computationally associated with the expression of CA VIII, CA X, and CA XI, to investigate their potential regulatory networks, enriched biological pathways, phenotypes, and disease associations. . The server works by considering the Gene identifiers as names in input and determines the respective TFs from available databases and conducted research studies in form of weighted biological networks which present the potential TFs rank wise. We also identified the specific cancer cell lines and tissue-specific expression sites for the relevant transcription factors (TFs), allowing us to pinpoint their precise sites of action and the cancer cell lines potentially targeted by the CAs influenced by these TFs. Additionally, we constructed a coexpression network for the genes corresponding to the three CAs, identifying the TFs and coexpressed genes associated with these CAs. This analysis enabled us to explore the diseases, biological pathways, and phenotypes linked to the activity of these genes and CAs. Furthermore, we determined the gene expression levels of these coexpressed genes across various tissues and organs, particularly in the context of cancer development, using data from microarray and RNA-Seq analyses. We utilized the GSEA and MSigDB servers [https://www.gsea-msigdb.org/] to generate heatmaps related to cancer expression for gene sets, which allowed us to visualize the upregulation and downregulation of specific genes within the associated biological pathways. We also extracted the set of various chemicals and drugs corresponding to the mechanistic connection to the TFs, respectively, that could have potential therapeutic as well as toxic responses against the disease-based phenotypes. In the further sections of the manuscript, we have performed an analysis of these specific drugs by determining their morphological and pharmacological properties to assess their similarity within the biological pathways. Therefore, by implementing this specific step, we were able to screen out the specific TFs and their respective biological parameters that were potentially related to the overall expression of CA VIII, CA X, and CA XI within the pathways of neurological and cancer-related conditions.

### 2.5. RASAR Model Generation

From the previous section, we obtained the set of drugs candidates that were mechanistically connected to the TFs corresponding to CA VIII, CA X, and CA XI, collectively. Hence, in this section, we utilized data mining approaches using drug and chemical databases like PubChem [https://pubchem.ncbi.nlm.nih.gov/] and Drugbank [https://go.drugbank.com/] to determine which of these chemicals were categorized into therapeutic (drug) and toxic (non-drug) candidates, respectively, depending on the information available from lab-based experiments and pharmacological data. Therefore, we created two different sets separating the therapeutic chemicals and toxic components in each list, where we performed a separate set of analyses involving the determination of substructure and PubChem-based molecular fingerprints from the PADEL Descriptors package [http://yapcwsoft.com/dd/padeldescriptor/]. Additionally, we also calculated the pharmacokinetic properties corresponding to the information related to ADMET parameters from the SwissADME server [http://www.swissadme.ch/index.php], which gave us an overall idea regarding both the structural information and the pharmacological information, which plays an integral part in drug development and screening processes. Following this step, we constructed a chemical network in form of a similarity matrix heatmap using Pearson correlation techniques to analyze the similarity among the chemical candidates from the therapeutic and toxic lists, respectively, considering their structural and pharmacokinetic properties. Hence, from the generated correlation heatmaps, we identified therapeutic and toxic compounds with similar structural and pharmacokinetic properties. These similarities provide a computational basis for prioritizing compounds with shared chemical characteristics and potential biological relevance for future experimental investigation. . Chemicals or drugs with similar structural and pharmacokinetic properties may exhibit similar biological activities and potential mechanisms of action (MoA). Consequently, these compounds may be computationally prioritized to investigate their potential associations with biological pathways, phenotypes, and transcription factor- or co-expressed gene-associated regulatory networks involving CA VIII, CA X, and CA XI, Following this, we developed a read-across QSAR model (RASAR) using machine learning (ML) techniques using Python programming and the scikit-learn library [https://scikit-learn.org/stable/index.html] to deploy a multi-model-based prediction algorithm that would cluster the therapeutic and toxic chemicals based on their structural and pharmacokinetic properties, respectively.

We employed a multi-model approach that utilized the KMeans clustering algorithm to group chemical candidates based on distance calculations by comparing the differences among various data points. Using the Euclidean distance method, we determined that the smaller the distance between chemicals, the more similar they are, and vice versa. This approach allowed us to successfully cluster therapeutic and toxic chemicals, which enabled the development of an automated machine learning (ML) model using the K Nearest Classifier (KNC). This model can assign unknown chemicals and drug candidates to clusters based on their structural and pharmacokinetic properties. We also designed a detailed workflow to analyze a set of chemical candidates for their therapeutic and toxic potential by clustering their morphological and pharmacological properties. The PCA-transformed molecular fingerprint dataset was divided into training (80%) and testing (20%) subsets in order to evaluate the stability and repeatability of the generated cluster assignments. The cluster labels derived from the clustering analysis were then used to train a K-Nearest Neighbours (KNN) classifier. Grid search was used to optimise hyperparameters by analysing various nearest neighbour counts (k = 3, 5, 7, 9, and 11) and weighting schemes (uniform and distance-based weighting). Five-fold cross-validation was used in the model selection process to reduce possible overfitting and enhance generalisability. Accuracy scores, classification reports, and confusion matrix analyses were used to assess the predictive performance of the optimal model after it was applied to the independent test set. Additionally, PCA-based scatter plots were created to show the distribution and separation of clusters based on molecular substructure fingerprints, and cross-validation accuracy trends were displayed across parameter combinations. .We then developed an ML-based predictive model to classify novel or unknown compounds into therapeutic or toxic classes based on their specific properties, comparing them with our trained model. This constructed model is particularly relevant for identifying drugs that may modulate transcription factors (TFs) computationally associated with CARP regulatory networks involved in neurological and cancer-related conditions. It does so by identifying compounds that may modulate transcription factor-associated regulatory networks linked to CA VIII, CA X, and CA XI, as evident from their functional enrichment in biological pathways associated with cancer development and the occurrence of neurological diseases.

## 3. Results and Discussion

As mentioned in the previous section, we conducted an end-to-end analysis to determine the biological expression of CA VIII, CA X, and CA XI, to understand their involvement in cancer and neurological development. We focused on their modulated enrichment along biological pathways, which are collectively influenced by the action of several transcription factors (TFs) and other co-expressed genes that play a crucial role in regulating the expression and associated biological networks of these CARPs, which are initially inactive due to conformational changes in their amino acid residues. Therefore, we conducted an extensive study aimed at developing drug discovery strategies to screen chemical candidates with potential inhibitory effects on suppressing the abnormal expression of TFs. By preventing the abnormal expression of CAs and their coexpressed genes, this approach provided valuable insights into potential treatment methods for cancer and neurological disorders. Additionally, we identified the most significant cell lines and tissues where the TFs associated with cancer and neurological disease development were localized. This analysis identified computational associations among TFs, CARPs, and enriched biological pathways, providing candidate regulatory networks that may be involved in cancer and neurological disorders. These findings offer a framework for future experimental studies to investigate the biological roles of these regulatory interactions. . By employing various clustering techniques and conducting frequency analysis of the most correlated CAs, we identified a specific set of biological parameters, including biological pathways and phenotypes, which were significantly expressed. These findings could serve as potential biomarkers for detecting the development of conditions associated with cancer and neurological disorders.

To further support the biomarker concept, we proposed a chemical analysis model designed to analyze therapeutic and toxic chemicals. This model could be used to either detect and treat biomarkers associated with disease development or identify chemicals that promote the progression of phenotypes related to biomarker expression along disease pathways. Using machine learning (ML) approaches, we developed a RASAR model to create a predictive algorithm capable of classifying unknown chemicals and drugs into therapeutic categories based on their structural and pharmacokinetic parameters. These parameters are linked to the mechanisms of action (MoA) associated with the modulation of biological pathways influenced by the expression of CAs through their respective TFs. In the following sections, we present the detailed workflow and the results obtained from our experiments.

### 3.1. Biological Network Analysis of CA VIII, X, XI

**Figure 1.**
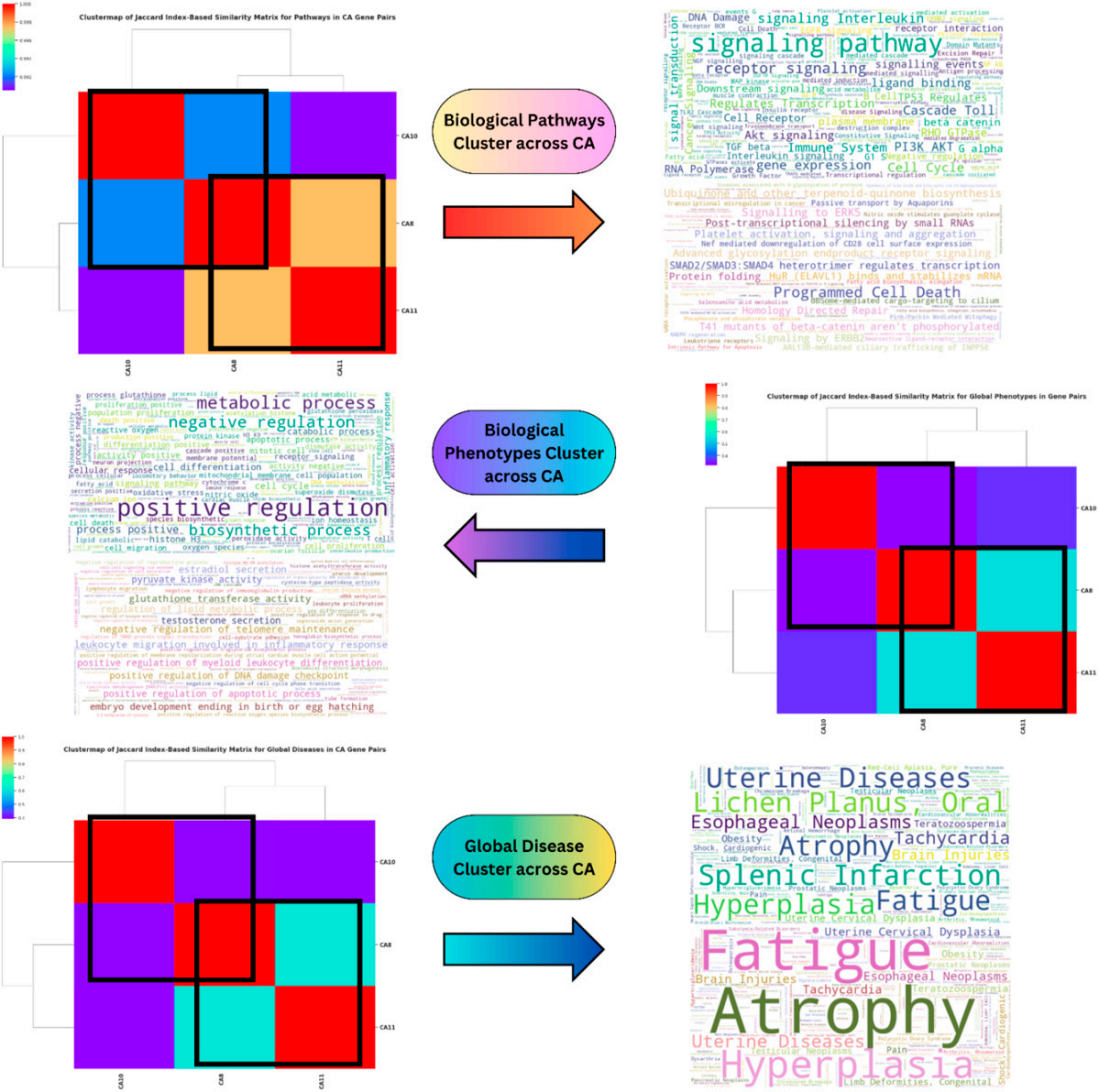
Jaccard Index (JI) clustermaps to analyze the global category of diseases, pathways and phenotypes associated with CA VIII, CA X, and CA XI

**Figure 2.**
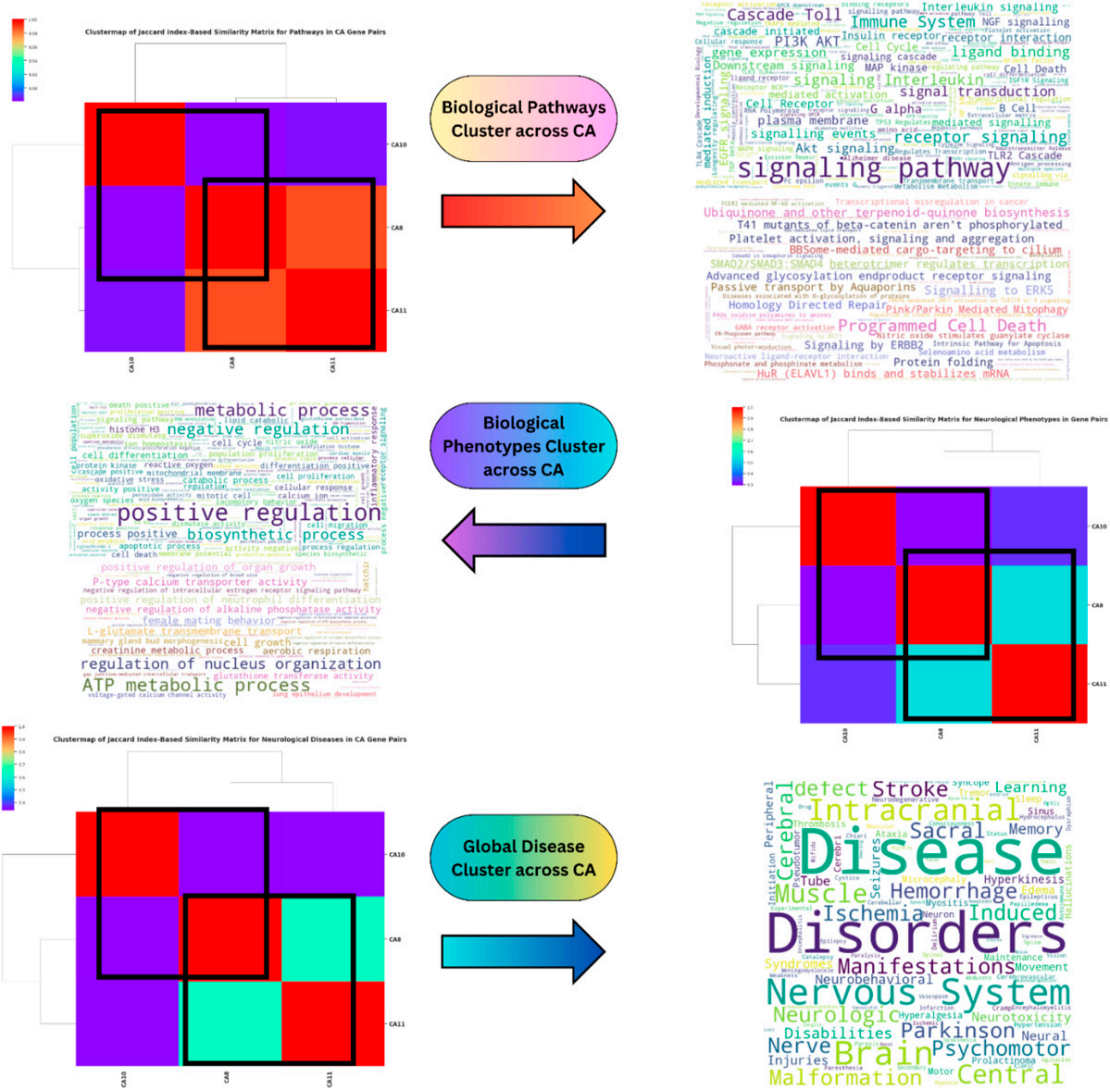
Jaccard Index (JI) clustermaps to analyze the neurological category of diseases, pathways, and phenotypes associated with CA VIII, CA X, and CA XI

Using the methodology described earlier, we performed a similarity matrix analysis on the CA dataset, which included information on biological pathways, disease categories, and phenotypes associated with CA VIII, CA X, and CA XI, By constructing Jaccard Index (JI) and hierarchical clustering (HC) heatmaps, we successfully analyzed the physiological similarities among the three CAs based on shared biological attributes. The HC method calculated distance metrics, with smaller dendrogram branches indicating greater similarity. We created separate JI and HC heatmaps for CA pairs, focusing on the pathways, disease categories, and phenotypes potentially linked to CA expression within the human immune system. To establish biological connections between CAs and disease development, we divided our analysis into two parts: one focused on neurological disorders and the other on global diseases, primarily cancer, involving carcinoma and neoplasms across multiple organs and tissues. We provided the graphical representations of the results obtained from our experimentation in the following figures (Figures 1 and 2), along with cluster representations across pairs of CA based on the mentioned biological parameters. Analyzing the representations, we could determine a stronger relationship shared between CA VIII and CA XI, respectively, with CA X having comparatively lesser similarity than others. The heatmaps of biological pathways, disease categories, and expressed phenotypes revealed a consistent correlation trend, providing strong evidence of significant similarity in the mechanisms and phenotype expression between CACA VIII and CA XI. This suggests a clear indication of potential disease development associated with the presence of these two CAs. Although CA X0 shows lower similarity scores compared to its counterparts, its involvement in disease development pathways remains significant and should not be overlooked. Following this, we also provided graphical representations, including word clouds, to illustrate the specific biological pathways, disease categories, and phenotypes within the clusters based on their frequency. In these word clouds, attributes with higher occurrences are displayed in larger fonts, while those with lower occurrences appear smaller. By combining the word cloud approach with the heatmaps, we gained valuable insights into the clusters formed using the Jaccard Index (JI). This allowed us to identify similar biological properties shared between the CAs and highlight specific attributes that occur more frequently within these clusters. From the provided representations, we observed that disease category distributions showed a higher frequency of multi-organ neoplasms and carcinoma within the global disease class, while the neurological disease class was characterized by various developmental and cognitive disorders localized around the CNS and spinal region. Similarly, our analysis of biological pathways revealed significant involvement of immune system-related and metabolism-related pathways, which play a key role in modulating biological reactions associated with cancer and neurological disease development. Additionally, examining the biological phenotypes across the CA pairs provided insights into the regulatory symptoms linked to cellular regulation and immune cell activation, which influences immune responses within the human immune system. Based on these findings, we identified the most frequent attributes for further analysis and hypothesized that these properties are likely to be expressed with the enrichment of the respective CAs within the human immune system.

### 3.2. Transcription Factors and Gene Co-Expression Network Analysis

**Figure 3.**
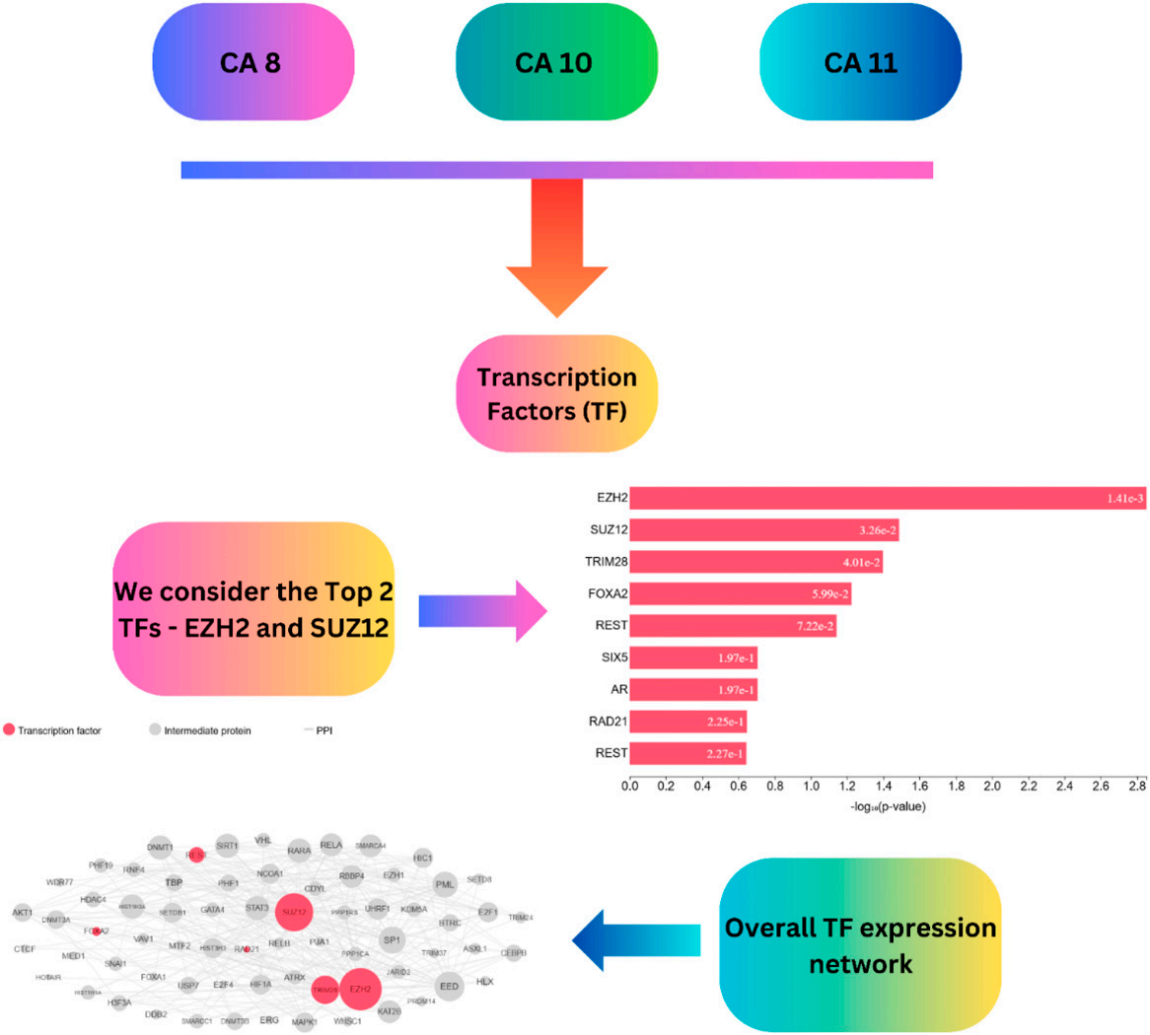
Determination of significant TFs associated with CA VIII, CA X, and CA XI along with their enrichment network

**Figure 4.**
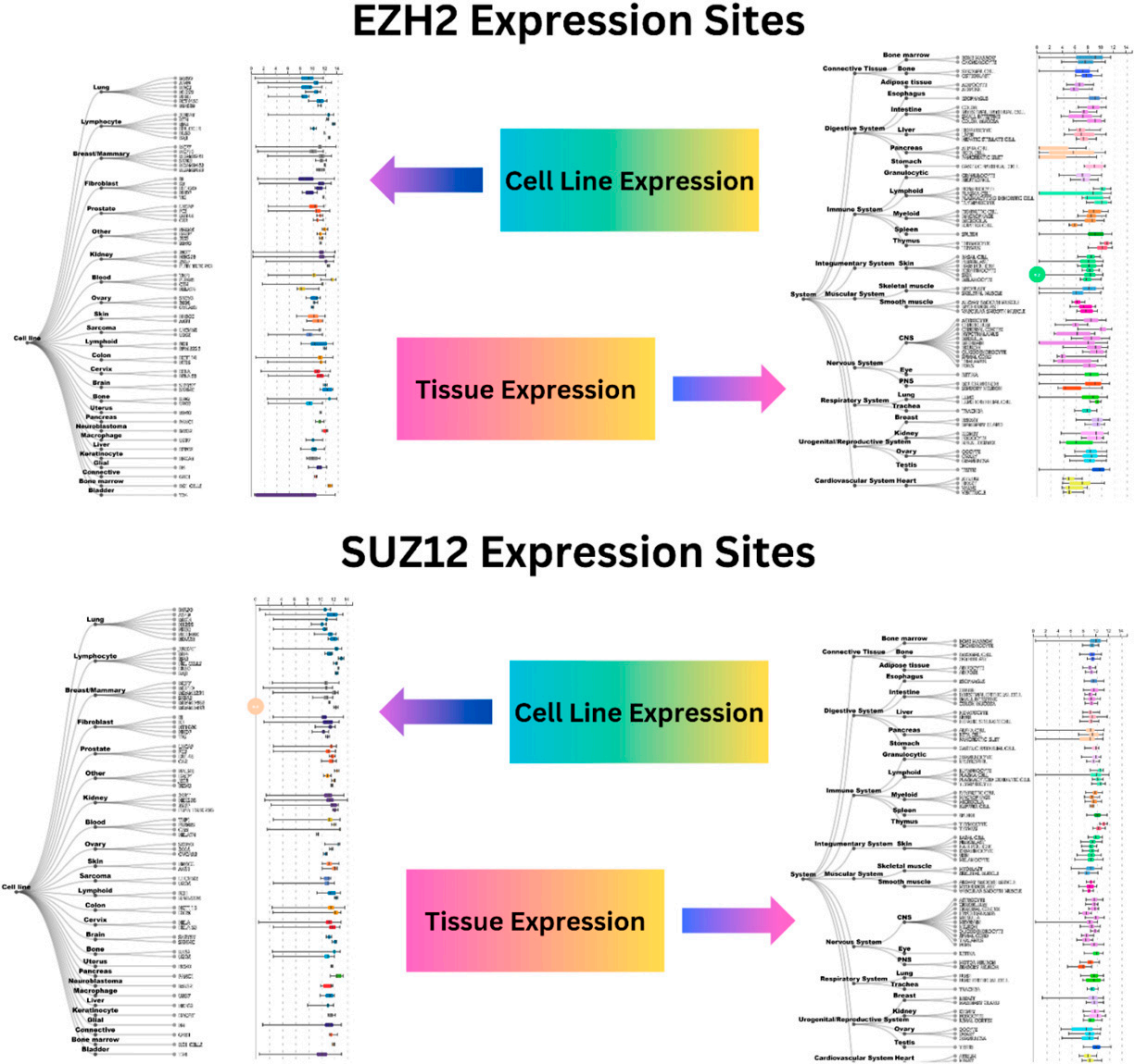
Tissue-based expression levels and cell line enrichment of the screened TFs within the CAs respectively

**Figure 5.**
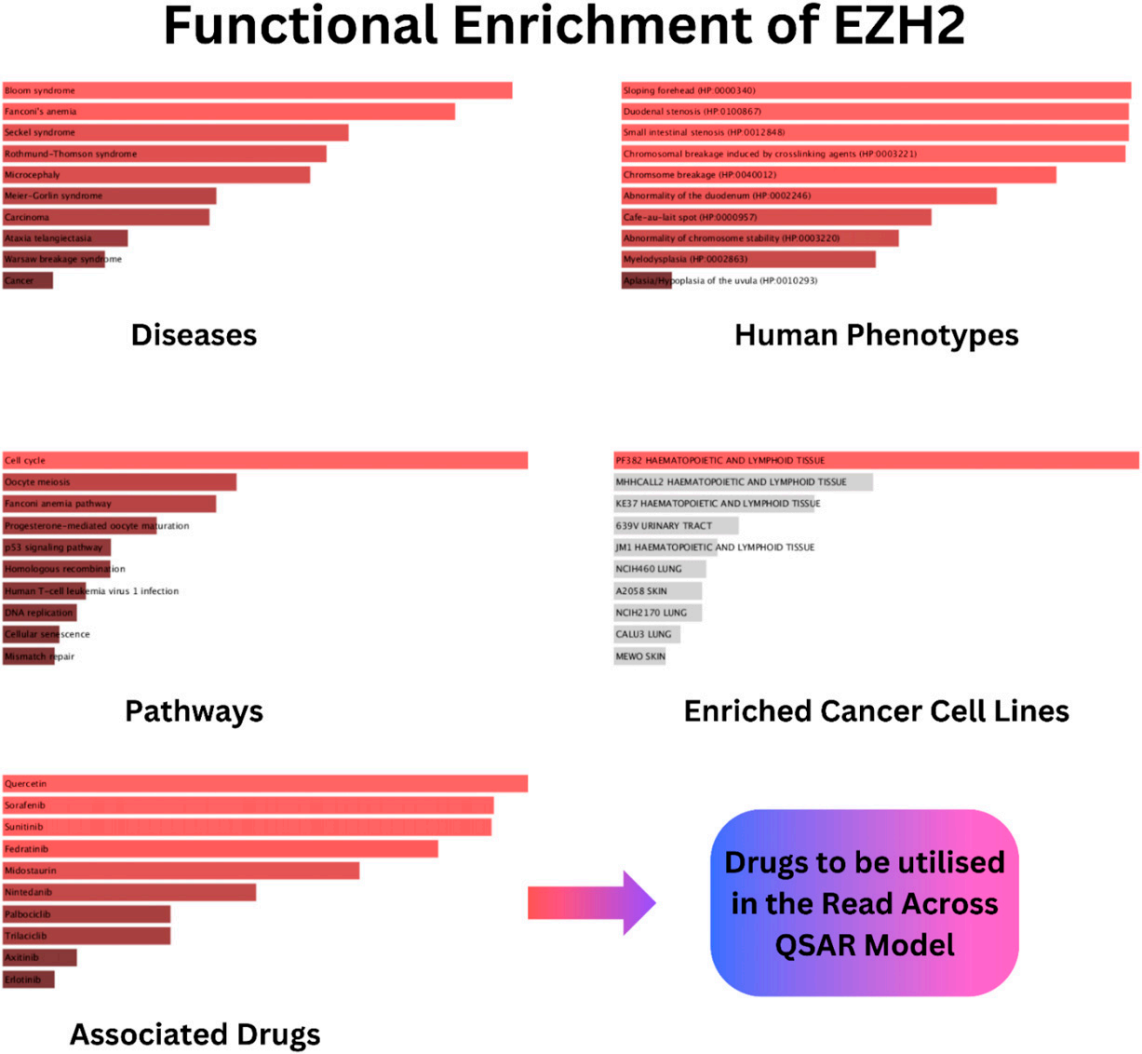
Functional enrichment analysis of TF: EZH2 and determination of potential drug candidates for QSAR modeling and chemical space analysis

**Figure 6.**
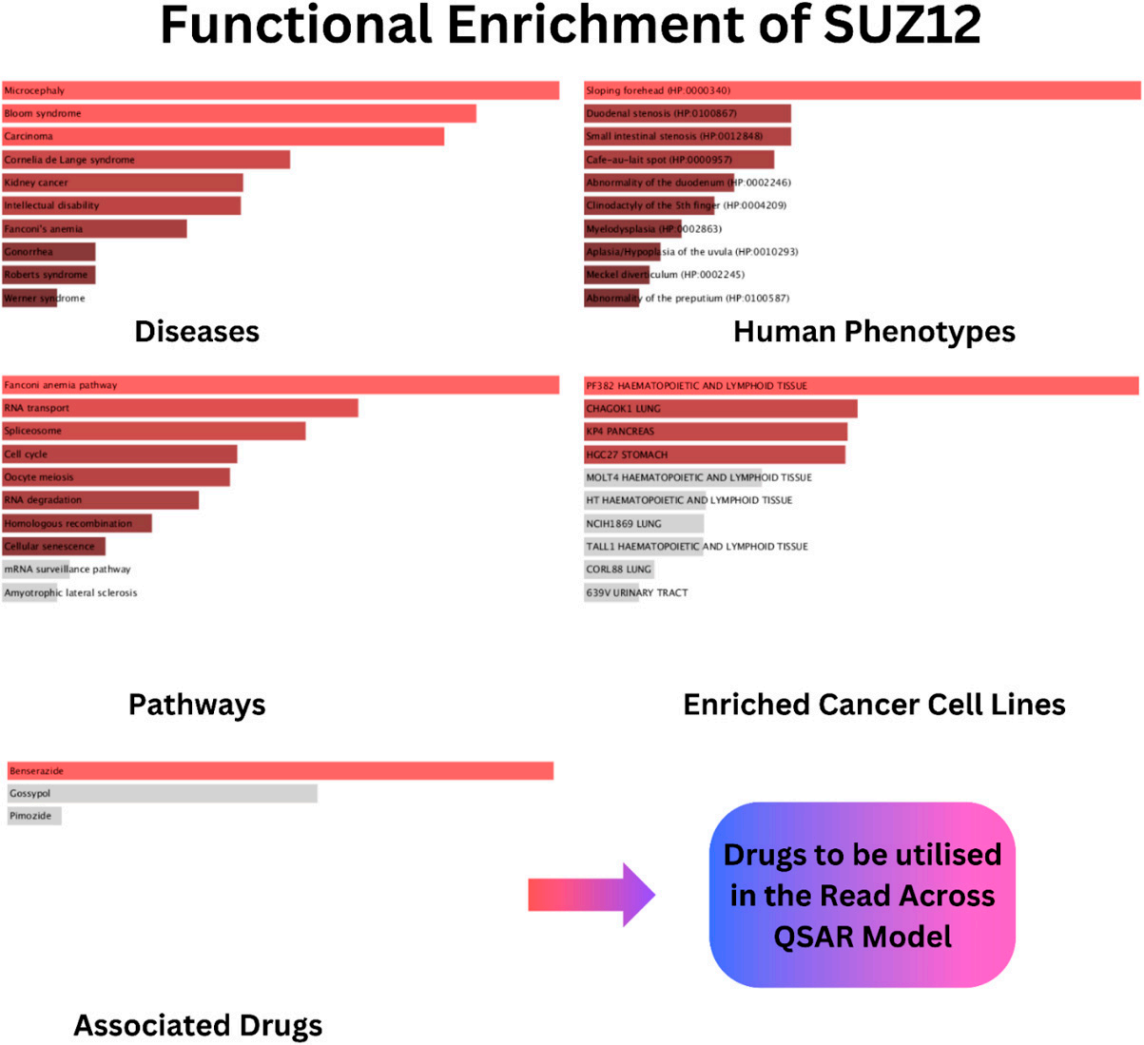
Functional enrichment analysis of TF: SUZ12 and determination of potential drug candidates for QSAR modeling and chemical space analysis

In the methods section, we analyzed the inactive nature of CA VIII, CA X, and CA XI, which is attributed to conformational changes caused by the positions of amino acid residues. Since CA VIII, CA X, and CA XI are catalytically inactive carbonic anhydrase-related proteins (CARPs), we investigated transcription factors (TFs) and co-expressed genes that are computationally associated with their expression and potential regulatory networks. We hypothesized that coordinated expression of CARPs with specific TFs and co-expressed genes may be associated with biological pathways and phenotypes linked to neurological disorders and cancer. To identify these associations, we constructed graph-based biological networks that enabled the prioritization of enriched pathways, phenotypes, and regulatory relationships across the three CARPs (Supplementary S1–S4). Additionally, in the following representations (Figures 3-6), we present the results obtained from the identification of TFs computationally associated with CARP expression. Additionally, we also included the specific biological diseases, pathways, and phenotypes, along with the tissue- and cell-line-specific expression graphs of the TFs within the human immune system. Analyzing the results, we could observe the two specific TFs, namely EZH2 and SUZ12, which corresponded to CA VIII and CA XI, respectively, hence following a similar trend to JI maps, where we had also observed shared similarity between the specific CAs. We also conducted an in-depth analysis of EZH2 and SUZ12 to evaluate their roles in various biological diseases, focusing primarily on cancer-related symptoms and certain neurological disorders. Our analysis identified specific biological pathways and phenotypes associated with these proteins, which were predominantly concentrated in areas such as cell signaling, immune cell modulation, and cancer pathways. The phenotypes identified included chromosomal defects, physical deformities, and aplasia or hypoplasia of tissues affected by cancer or neurological disorders. Additionally, our analysis of tissue expression and cell line determination for the mentioned TFs revealed a predominant presence in neurological tissues and pulmonary cells, with cancer cell lines primarily concentrated in these areas and other organs. This finding provided valuable insights into the potential for cancer development across multiple tissues and organs, underscoring the need for developing drugs and chemical compounds that could inhibit TF expression. Furthermore, our representations identified potential drugs and chemical candidates that could serve as therapeutic agents to modulate the activity of these TFs, thereby controlling the negative regulatory effects of CAs in cancer and neurological disorders. We applied similar analytical approaches to assess the TFs associated with each CARP individually, as detailed in the supplementary files (Supplementary S5–S13), which revealed comparable expression patterns and associations with biological pathways and phenotypes related to cancer and neurological disorders. The TFs associated with each of the CAs and their co-expressed genes are represented in the table below for consideration (Table 2). We also provided graphical representations (Supplementary S14-S16) that illustrate the connections between CAs and their co-expressed genes, which are potential targets of TF-associated drugs. These representations include multi-organ cancer expression data obtained from various RNA-Seq and microarray analyses, offering detailed insights into the transcriptomic expression of these specific genes across different tissues where cancer development may occur. The graphical data also indicate which genes are upregulated or downregulated, based on the color coding. Through this analytical workflow, we identified computational associations between the CARPs, transcription factors, co-expressed genes, and enriched biological pathways, providing candidate regulatory networks and biomarkers potentially associated with cancer and neurological disorders.

**Table 2.** Transcription Factors (TFs) Associated With Each Carbonic Anhydrase-Related Protein (CARP) and Their Corresponding Co-Expressed Genes

CA type	TF representations
CA VIII (8)	ESR1, NFE2L2
CA X (10)	CTCF, ZBTB7A
CA XI (11)	PPARG, RAD21

### 3.3. Development of RASAR Models

**Figure 7.**
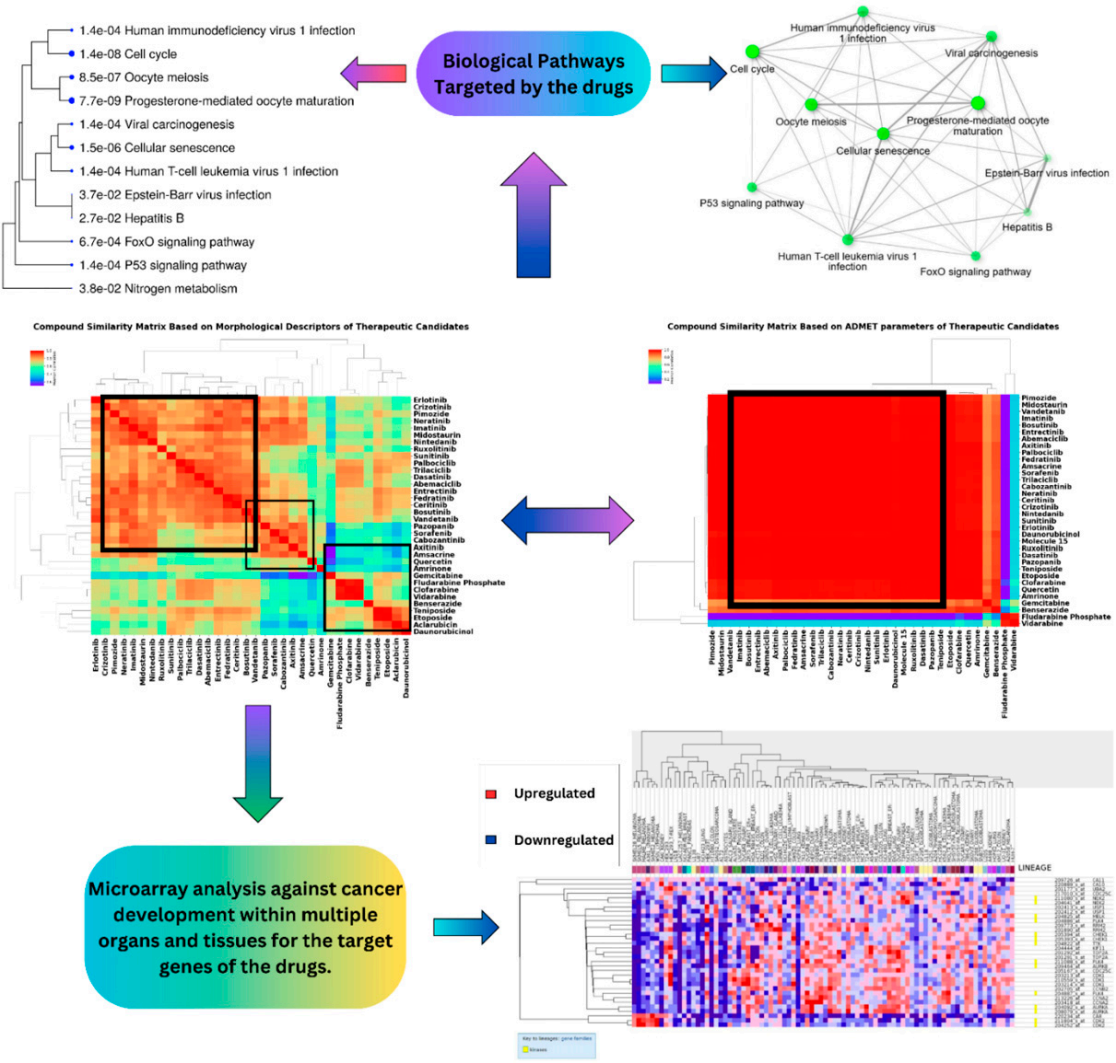
Chemical space analysis of the therapeutic drugs candidates associated with the screened TFs based on their morphological and pharmacological properties. Additionally, we determined the target biological pathways related to the MoA of the chemicals along with their microarray analysis to assess the target gene expression levels across multi-organ cancer cell lines

**Figure 8.**
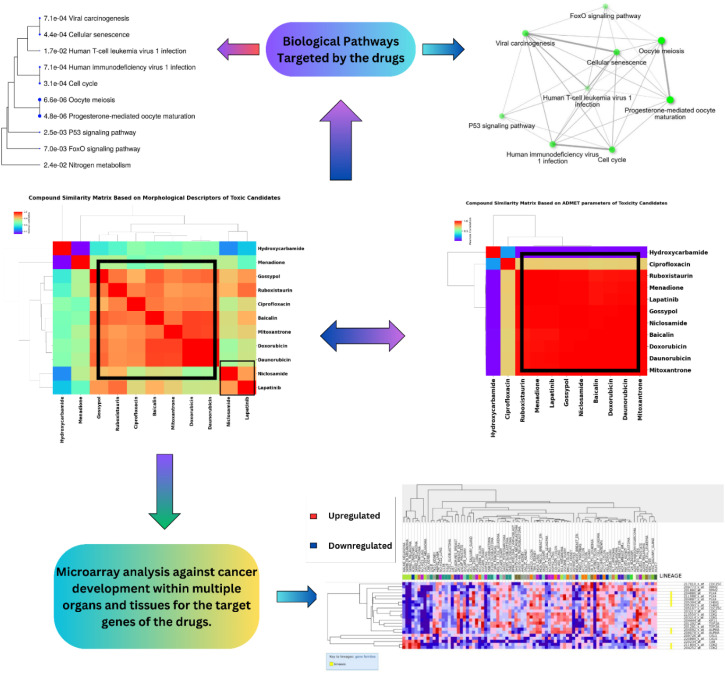
Chemical space analysis of the toxic drugs and chemical candidates associated with the screened TFs based on their morphological and pharmacological properties. Additionally, we determined the target biological pathways related to the MoA of the chemicals along with their microarray analysis to assess the target gene expression levels across multi-organ cancer cell lines

**Figure 9.**
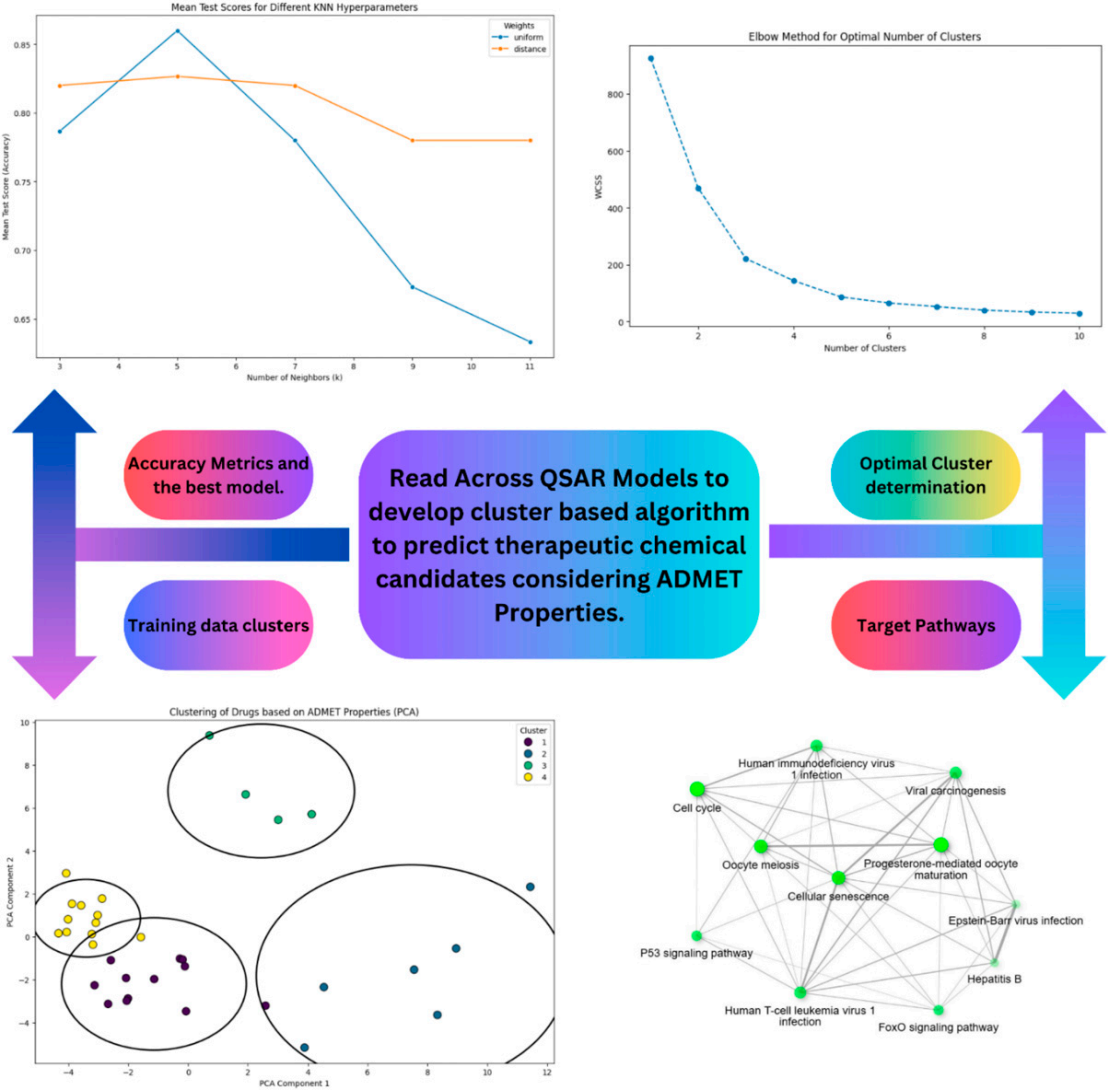
RASAR model to analyze the therapeutic drugs based on their pharmacological parameters and analyze the clustering based on the similarity of ADMET parameters among the chemical candidates. Additionally, we also included the accuracy metrics and model selection graph with the target biological pathways potentially involved with the MoA of the respective drugs

**Figure 10.**
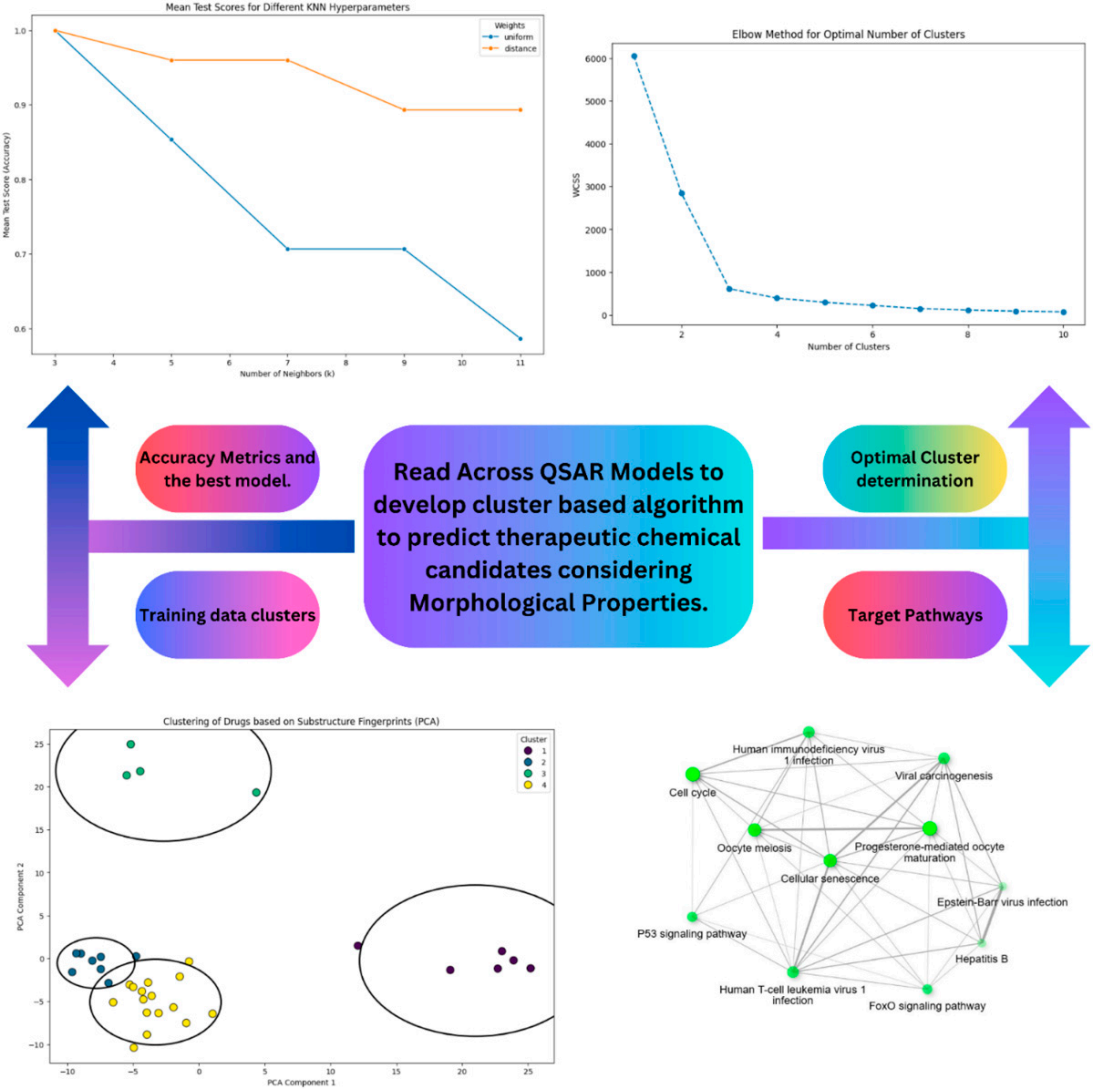
RASAR model to analyze the therapeutic drugs based on their morphological parameters and analyze the clustering based on the similarity of structural parameters among the chemical candidates. Additionally, we also included the accuracy metrics and model selection graph with the target biological pathways potentially involved with the MoA of the respective drugs

From the analysis involving TF determination, we screened out the specific drugs candidates associated with inducing inhibitory effects against the negative impact of those TFs in the expression of CA and its co-expressed genes, which ultimately lead to the development of cancer conditions and neurological disorders. Hence, to extract the specific drugs that modulate the activity of the TFs associated with CA VIII, CA X, and CA XI, respectively, we curated the drugs into a separate dataset and computed the molecular descriptors, specifically concentrating on the physicochemical descriptors and substructure fingerprints, which contain information regarding the overall conformational information related to molecular interactions, scaffold localizations, atomic and molecular components, etc. Additionally, we also calculated the pharmacokinetic parameters for the drugs by determining ADMET properties. We were able to understand how these specific drugs would act in a pharmacological environment through certain properties like blood brain barrier (BBB) permeability, solubility, molecular weights, gastrointestinal absorption property, etc. Thus, combining the mentioned two parameters, we successfully obtained a wide spectrum of information related to both structural and functional properties, which would provide rigid evidence regarding the development of drugs to deal with disease development. Additionally, referring to information from drug databases as mentioned in the methods and materials section, we divided the drug dataset into therapeutic and toxic classes, respectively, depending on their MoA within the human immune system. Subsequently, we calculated the structural and pharmacokinetic descriptors for both sets of drugs and created a similarity index using Pearson correlation values. This analysis provided detailed information on the structural and functional similarities between chemical candidates from each dataset. Hence, this represents an important aspect of drug discovery and QSAR methodologies, as compounds with similar structural and pharmacokinetic properties may exhibit similar biological activities. Consequently, these compounds can be computationally prioritized for further experimental evaluation against disease-associated biological pathways. . The graphical representations denoting the mentioned methodology are shown in the following diagrams (Figures 7 and 8), and the clusters marked in black-bordered representations denote that the specific chemical candidates are potentially similar in their MoA and can potentially induce either therapeutic or toxic effects against the biological targets associated with disease development. We also developed an ML-assisted RASAR model, which would enable us to cluster the various sets of therapeutic chemical candidates together based on their structural and pharmacokinetic properties. This ML model further converts the clustering algorithm into a predictive tool. When presented with a novel chemical or drug with uncertain mechanisms, the model calculates its structural and pharmacokinetic properties and compares them with the trained therapeutic cluster of compounds. The model would then assign the unknown candidate within it, denoting a similar MoA with the therapeutic compounds. Hence, by deploying this end-to-end methodology, we would be able to perform virtual screening of various chemical candidates to determine their overall biological nature and understand whether they could be utilized in treating cancer and neurological disorders. From the provided representation below (Figures 9 and 10), we can observe the clusters formed across the therapeutic compounds, considering their structural and pharmacokinetic properties, respectively. We employed a multimodel machine learning methodology, combining KMeans clustering and KNC, to perform clustering based on distance-based calculations between the physiological properties of the drugs, while also developing a predictive model within each cluster.From the results obtained we can observe the performance of the predictive performance and robustness of the ML-assisted RASAR framework. The best K-Nearest Neighbours classifier was found using uniform weighting and k = 3. The final model’s overall prediction accuracy on the independent test dataset was 85.7%. The weighted-average precision, recall, and F1-scores from the performance evaluation were 0.75, 0.86, and 0.80, respectively. The stability of the identified therapeutic clusters was supported by confusion matrix analysis, which showed a high level of agreement between predicted and assigned cluster labels. Ultimately the distance-based clustering methodology was observed to have more weight in the overall development processes. By combining all the above approaches, we successfully developed a comprehensive computational human health model. This model enabled an end-to-end computational analysis to identify biological factors computationally associated with the expression of CA VIII, CA X, and CA XI in the context of cancer and neurological disorders. Additionally, it facilitated the prioritization of biological pathways, phenotypes, and candidate compounds that may serve as a foundation for future experimental studies and drug discovery efforts.

## 4. Conclusion

In conclusion, our manuscript presents a comprehensive workflow to analyze the catalytically inactive carbonic anhydrase-related proteins (CARPs), specifically CA VIII, CA X, and CA XI, and their potential roles in cancer development across various organs and in neurological diseases, including cognitive disorders, memory impairment, and motor neuron disorders. We explored the biological relationships between these inactive genes, focusing on the possible connections between CAs, transcription factors (TFs), and other co-expressed genes. These interactions may regulate the expression of CARPs and their associated regulatory gene networks, contributing to the enrichment of biological pathways potentially associated with the development of multiorgan cancers and neurological disorders. By identifying specific TFs and genes that may be associated with the expression of CARPs, we provide insights into the core biological pathways involved in disease phenotypes associated with cancer and neurological conditions. Through computational analysis of tissue-specific expression and enriched cell lines, we identified transcription factors that are computationally associated with CARP-related regulatory networks. Subsequently, we prioritized drugs and chemical compounds that may potentially modulate these transcription factor-associated networks for future experimental evaluation. Moreover, we proposed a computational human health model that employs morphological and pharmacokinetic descriptors to analyze drugs targeting these CAs. This approach enabled the development of a RASAR model, facilitating the creation of predictive models to categorize novel drugs based on their therapeutic nature by considering their physicochemical properties. By leveraging this analysis, we successfully developed a possible virtual-screening methodology to design drug molecules capable of detecting biomarkers associated with the progression of cancer and neurological diseases, effectively targeting the relevant genes, TFs, and pathways enriched during disease development. This computational workflow when integrated or validated via experimental approaches to analyse the CARPs against the cancer cell lines would enable future development for more advanced computational pipelines to explore other carcinogenic conditions.

Ultimately, as this is a computational workflow, there are certain limitations that require further experimental validation to assess the biological relevance of the findings. Because this study mostly relies on publicly accessible databases and computational analyses, it is susceptible to issues with database completeness, annotation bias, and the underlying assumptions of bioinformatics prediction tools. The inferred transcription factor–CARP regulatory relationships need to be validated experimentally because they are computational predictions. Furthermore, the generalisability of our findings may be limited because they were not verified in separate patient cohorts. Lastly, rather than providing proof of clinical efficacy, the RASAR and drug-similarity analyses offer computational prioritisation of possible therapeutic candidates; therefore, in vitro, in vivo, and clinical studies should validate their translational relevance.

## Supplemental Material

Supplemental material - Unveiling Carbonic Anhydrase VIII, X, XI Expression in Cancer and Neurological Diseases through integrated bioinformatics approaches Supplemental material for Unveiling Carbonic Anhydrase VIII, X, XI Expression in Cancer and Neurological Diseases through integrated bioinformatics approaches by Ratul Bhowmik, Rajarshi Ray, and Ashok Aspatwar in Bioinformatics and Biology Insights.

## Data Availability

The datasets used to develop the workflow for this manuscript, along with the Python script and obtained results, are provided in the following Zenodo repository: https://zenodo.org/records/12796528.

## Appendix

### Abbreviations

ADMET: Absorption, Distribution, Metabolism, Excretion, and Toxicity
CA: Carbonic Anhydrase
CARP: Carbonic Anhydrase-Related Protein
CTD: Comparative Toxicogenomics Database
DNA: Deoxyribonucleic Acid
GSEA: Gene Set Enrichment Analysis
GO: Gene Ontology
HC: Hierarchical Clustering
JI: Jaccard Index
KEGG: Kyoto Encyclopedia of Genes and Genomes
K-Means: K-Means Clustering Algorithm
KNN: K-Nearest Neighbours
ML: Machine Learning
MoA: Mechanism of Action
mRNA: Messenger Ribonucleic Acid
MSigDB: Molecular Signatures Database
PCA: Principal Component Analysis
QSAR: Quantitative Structure–Activity Relationship
RASAR: Read-Across Structure–Activity Relationship
ROC: Receiver Operating Characteristic
RNA-seq: RNA Sequencing
RT-PCR: Reverse Transcription Polymerase Chain Reaction
TF: Transcription Factor
X2K: Expression2Kinases
Zn^2+^: Zinc ion.
