# Supplementary material for: Unveiling Carbonic Anhydrase VIII, X, XI Expression in Cancer and Neurological Diseases Through Integrated Bioinformatics Approaches
Source: Bioinform Biol Insights. 2026 Aug 3;20:11779322261475822. doi: 10.1177/11779322261475822 (PMC13434096; doi:10.1177/11779322261475822)
Supplement: Supplementary file 1 [file sj-pdf-1-bbi-10.1177_11779322261475822.pdf]

Unveiling Carbonic Anhydrase VIII, X, XI Expression in Cancer and Neurological Diseases through integrated bioinformatics approaches

Ratul Bhowmik[1#], Rajarshi Ray[1#], and Ashok Aspatwar[1*]

[1]Faculty of Medicine and Health Technology, Tampere University, FI-33520 Tampere, Finland.

ratul.bhowmik@tuni.fi, rajarshi.ray@tuni.fi, ashok.aspatwar@tuni.fi

***Corresponding author**

Dr. Ashok Aspatwar,

ashok.aspatwar@tuni.fi

#These authors contributed equally to this manuscript

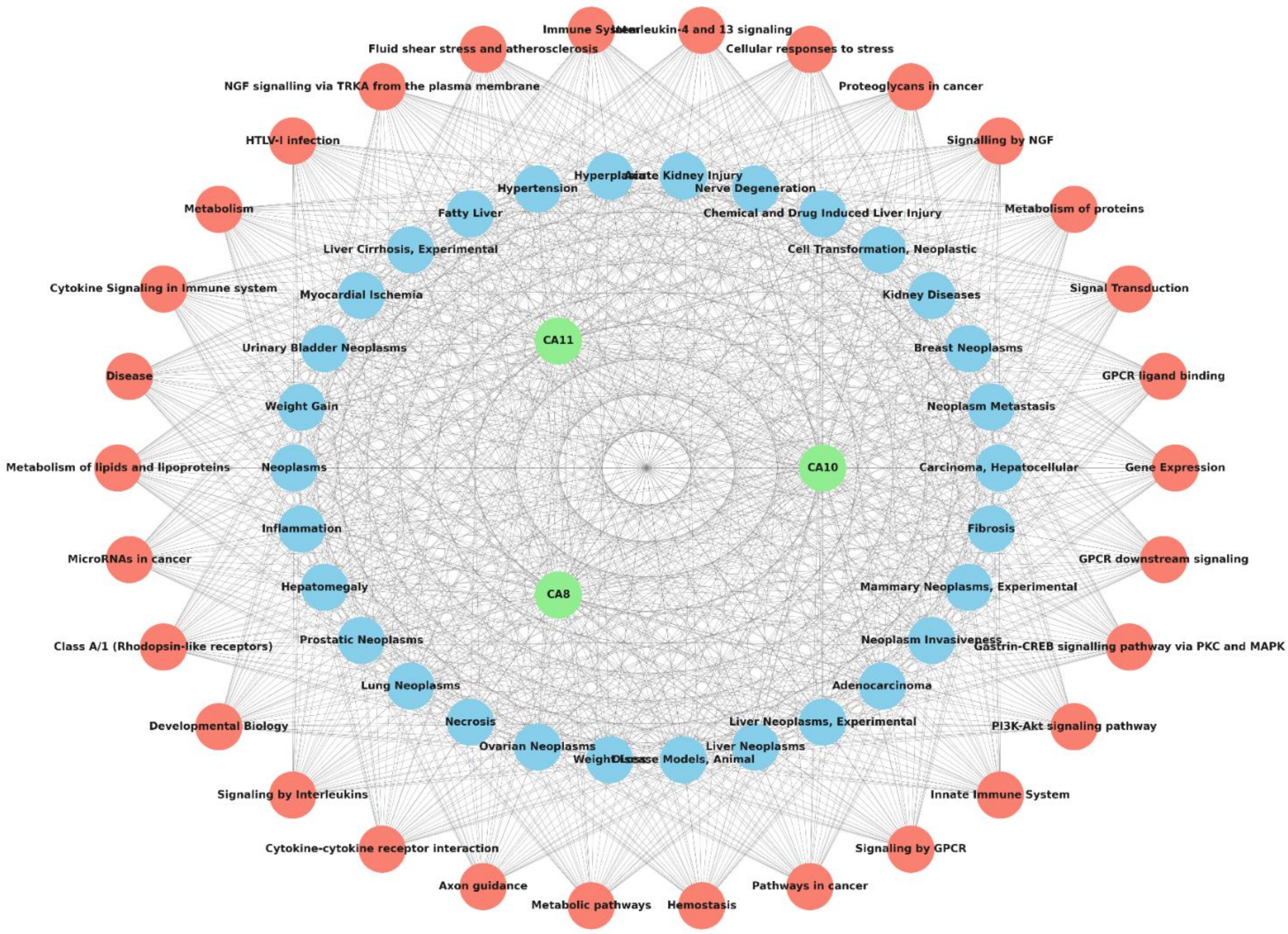

Figure S1 – Biological network representing the biological interactions among CA 8, 10 and 11 and Global category of diseases along with its respective biological pathways which are significantly enriched within its development.

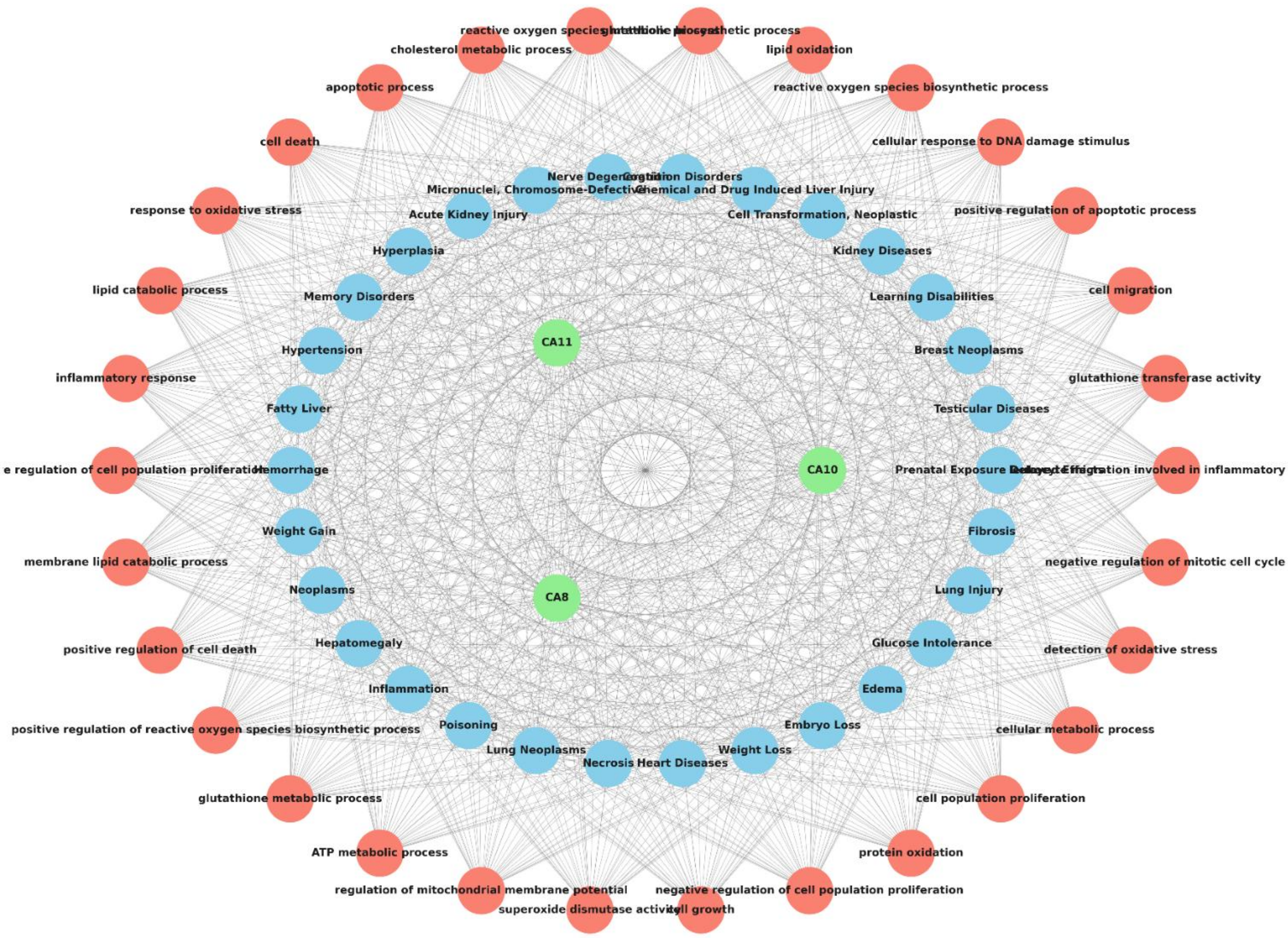

Figure S2 – Biological network representing the biological interactions among CA 8, 10 and 11 and Global category of diseases along with its respective biological phenotypes which are significantly expressed within its development.

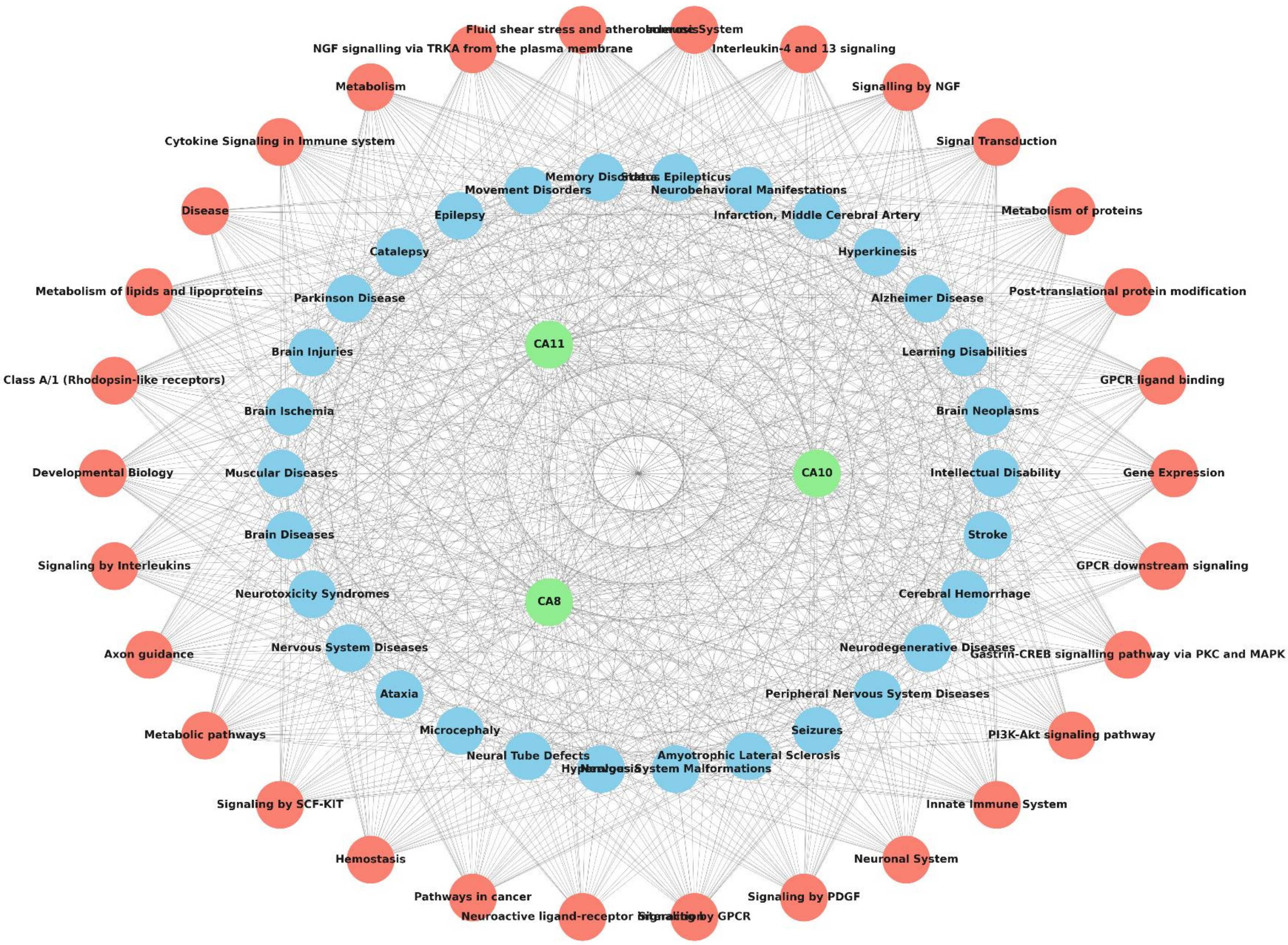

Fluid shear stress and atherosclerosis
Immune System
NGF signalling via TRKA from the plasma membrane
Interleukin-4 and 13 signaling
Metabolism
Signalling by NGF
Cytokine Signaling in Immune system
Signal Transduction
Disease
Metabolism of proteins
Metabolism of lipids and lipoproteins
Post-translational protein modification
Class A/1 (Rhodopsin-like receptors)
GPCR ligand binding
Developmental Biology
Gene Expression
Signaling by Interleukins
GPCR downstream signaling
Axon guidance
Gastrin-CREB signalling pathway via PKC and MAPK
Metabolic pathways
PI3K-Akt signaling pathway
Signaling by SCF-KIT
Innate Immune System
Hemostasis
Neuronal System
Pathways in cancer
Signaling by PDGF
Neuroactive ligand-receptor interaction
Signaling by GPCR
Memory Disorders
Status Epilepticus
Movement Disorders
Neurobehavioral Manifestations
Epilepsy
Infarction, Middle Cerebral Artery
Catalepsy
Hyperkinesis
Parkinson Disease
Alzheimer Disease
Brain Injuries
Learning Disabilities
Brain Ischemia
Brain Neoplasms
Muscular Diseases
Intellectual Disability
Brain Diseases
Stroke
Neurotoxicity Syndromes
Cerebral Hemorrhage
Nervous System Diseases
Neurodegenerative Diseases
Ataxia
Peripheral Nervous System Diseases
Microcephaly
Seizures
Neural Tube Defects
Amyotrophic Lateral Sclerosis
Hyperalgesia
Nervous System Malformations
CA11
CA10
CA8

Figure S3 – Biological network representing the biological interactions among CA 8, 10, and 11 and the Neurological category of diseases along with its respective biological pathways which are significantly enriched within its development.

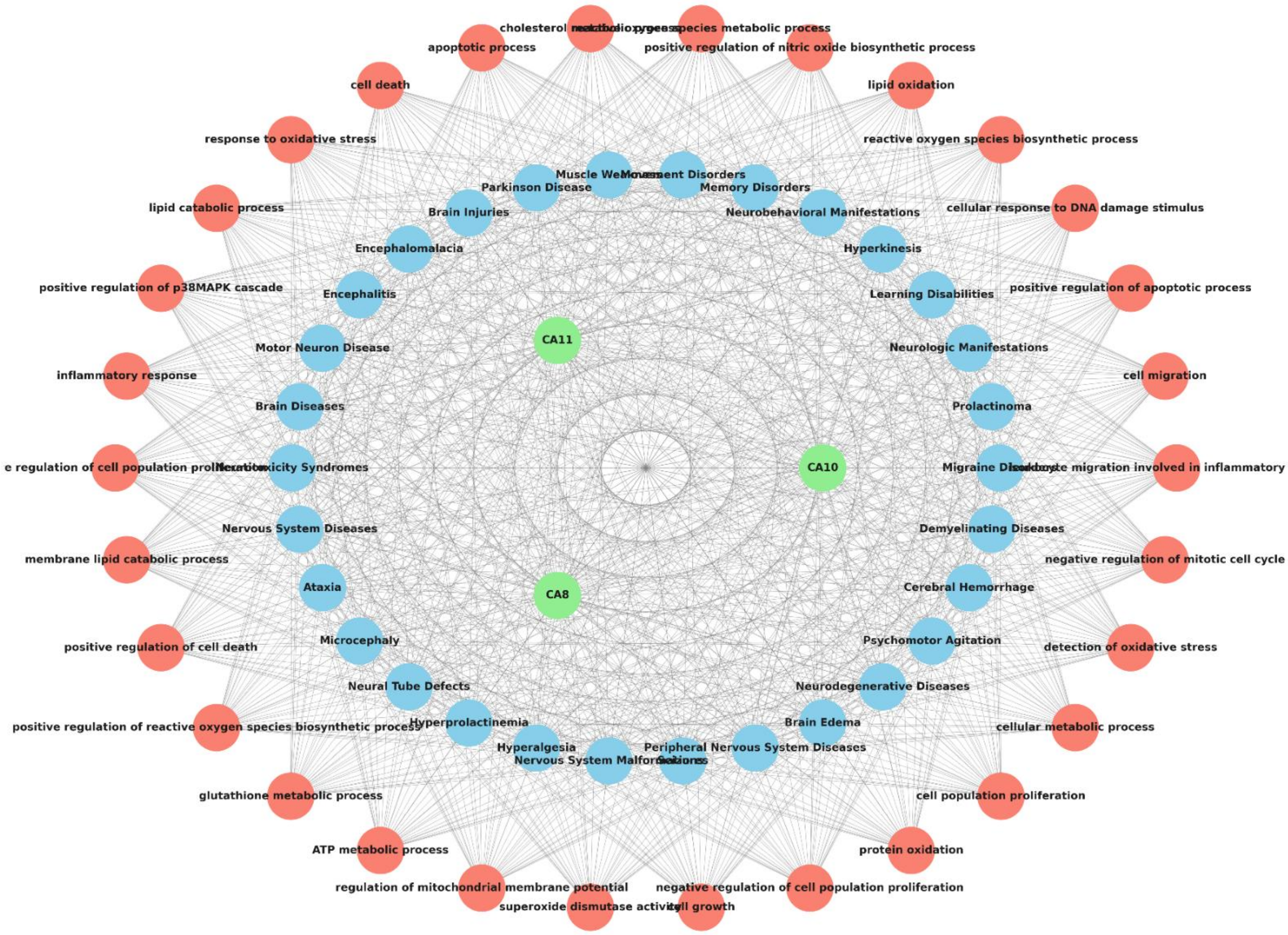

Figure S4 – Biological network representing the biological interactions among CA 8, 10, and 11 and the Neurological category of diseases along with its respective biological phenotypes which are significantly expressed within its development.

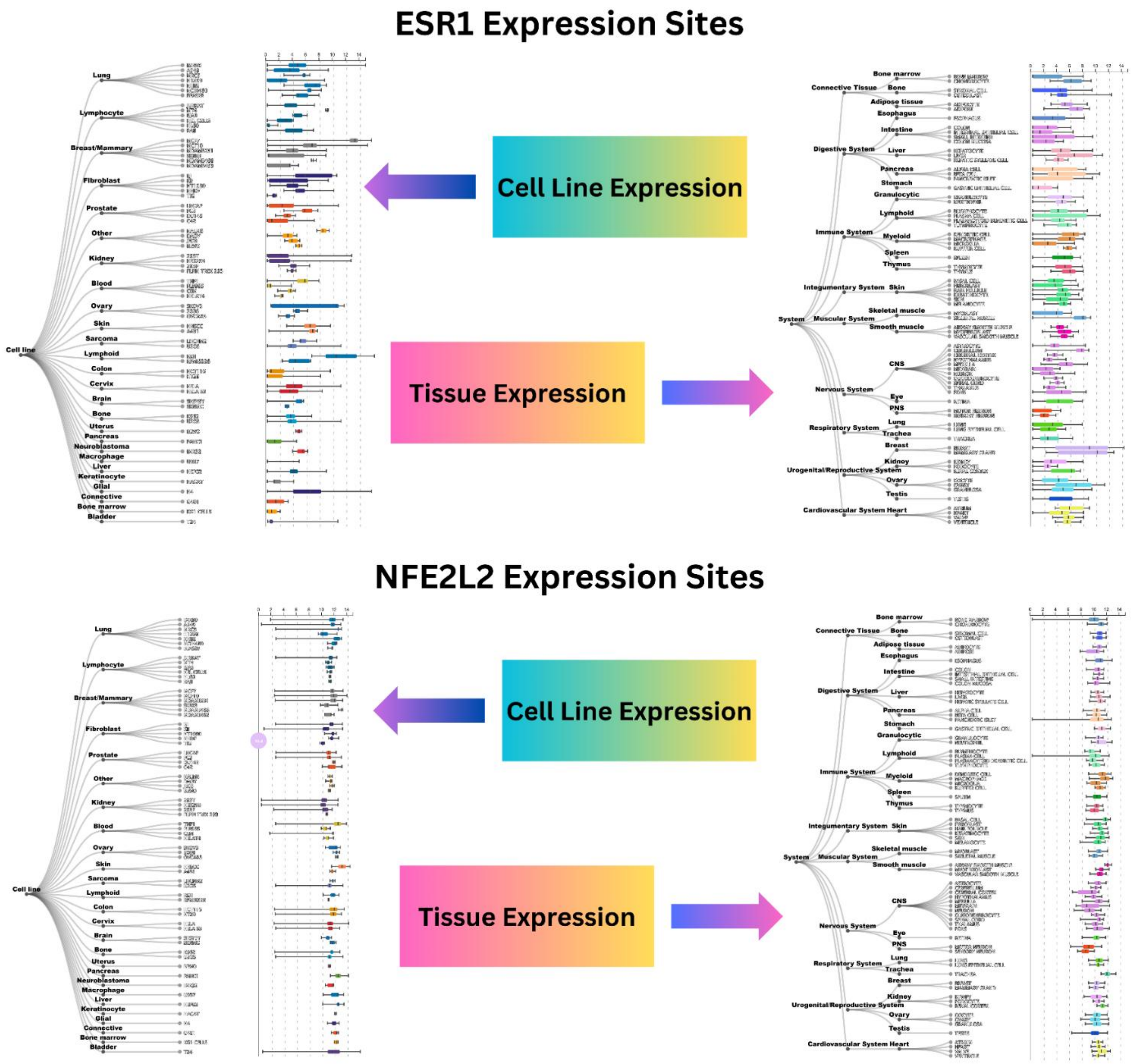

Figure S5 – Tissue-based expression levels and cell line enrichment of the screened TFs (ESR1 and NFE2L2) within the CA8 respectively.

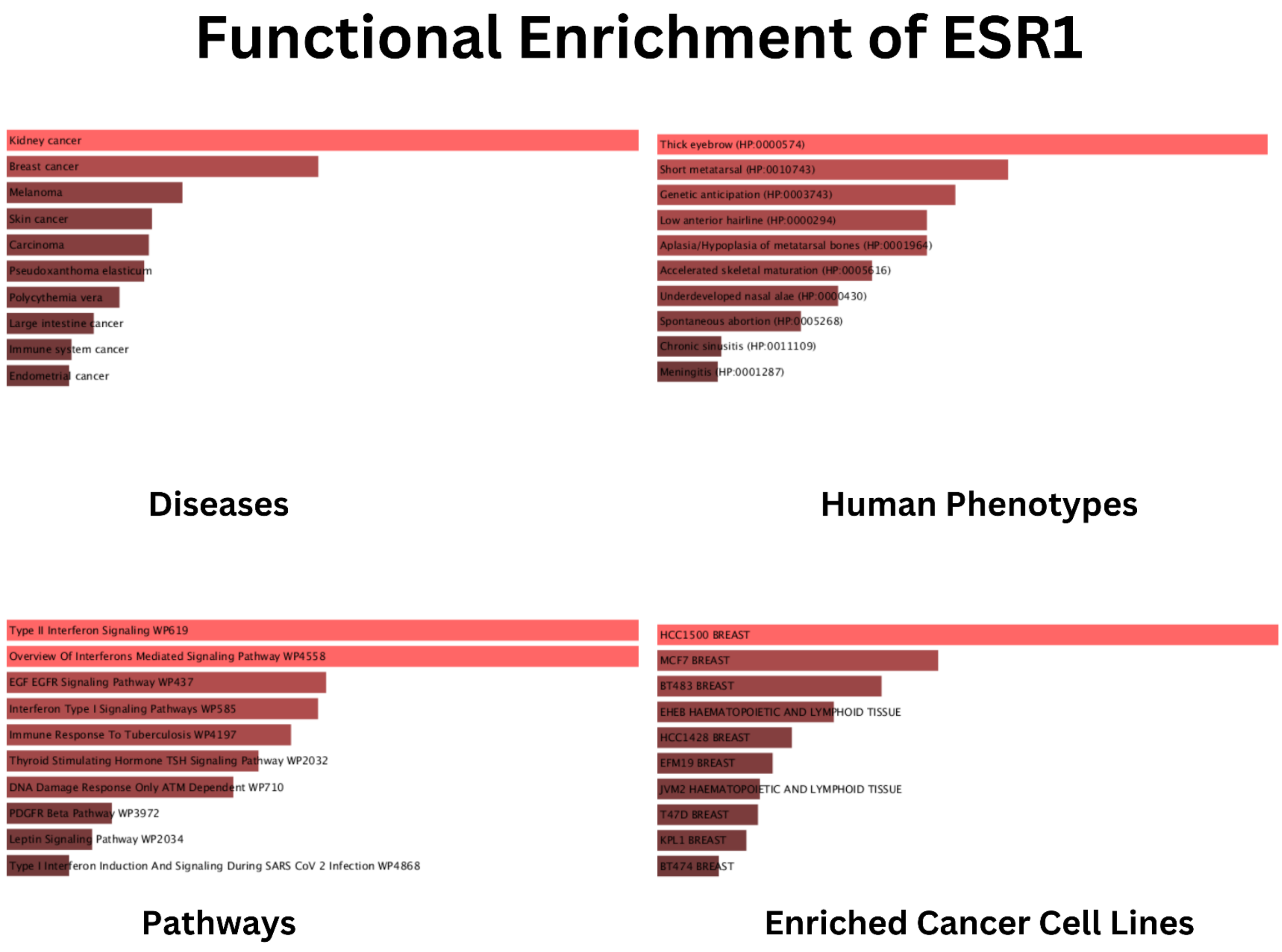

Figure S6 – Functional enrichment analysis of TF: ESR1 within CA8

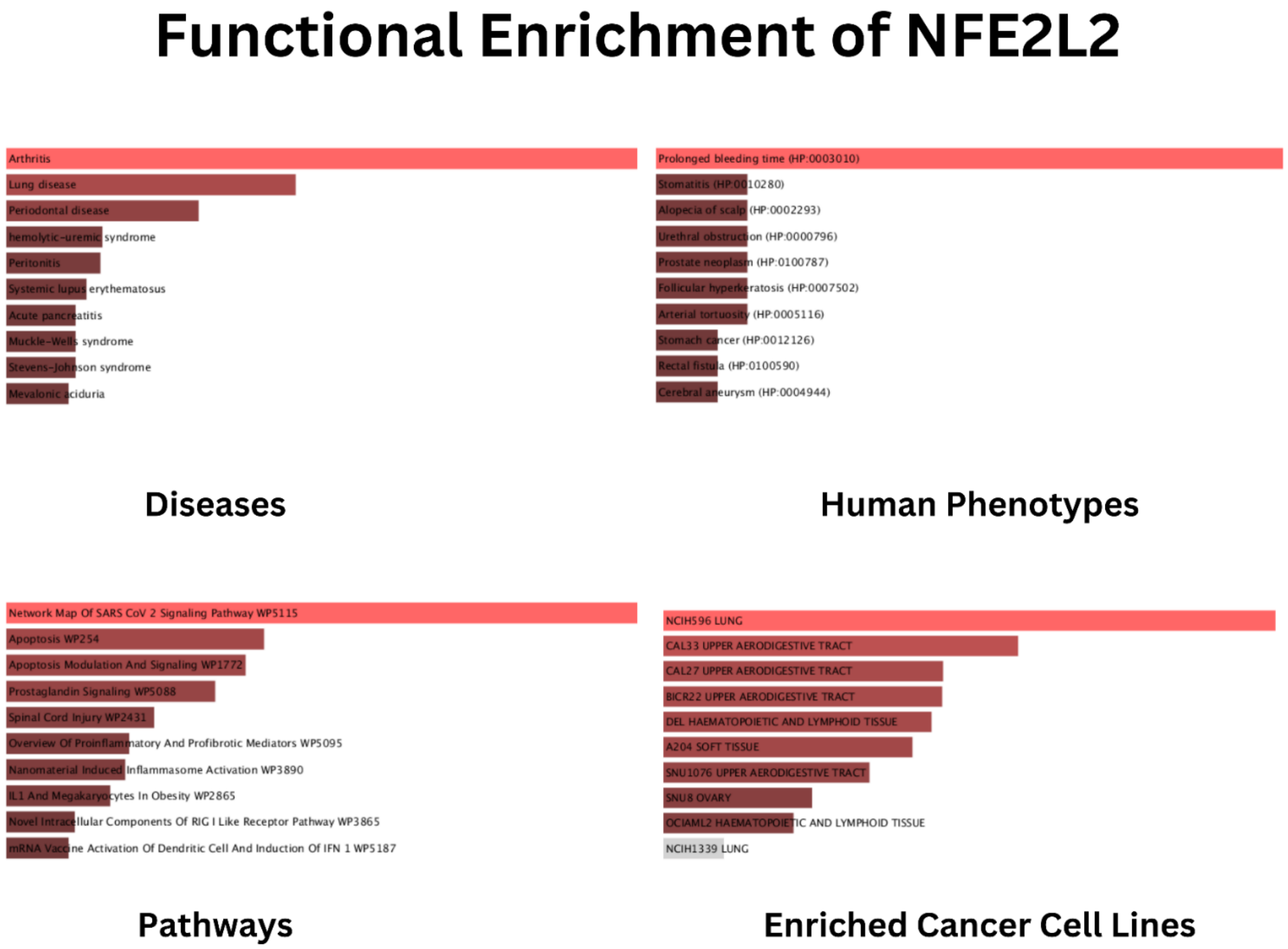

Figure S7 – Functional enrichment analysis of TF: NFE2L2 within CA8

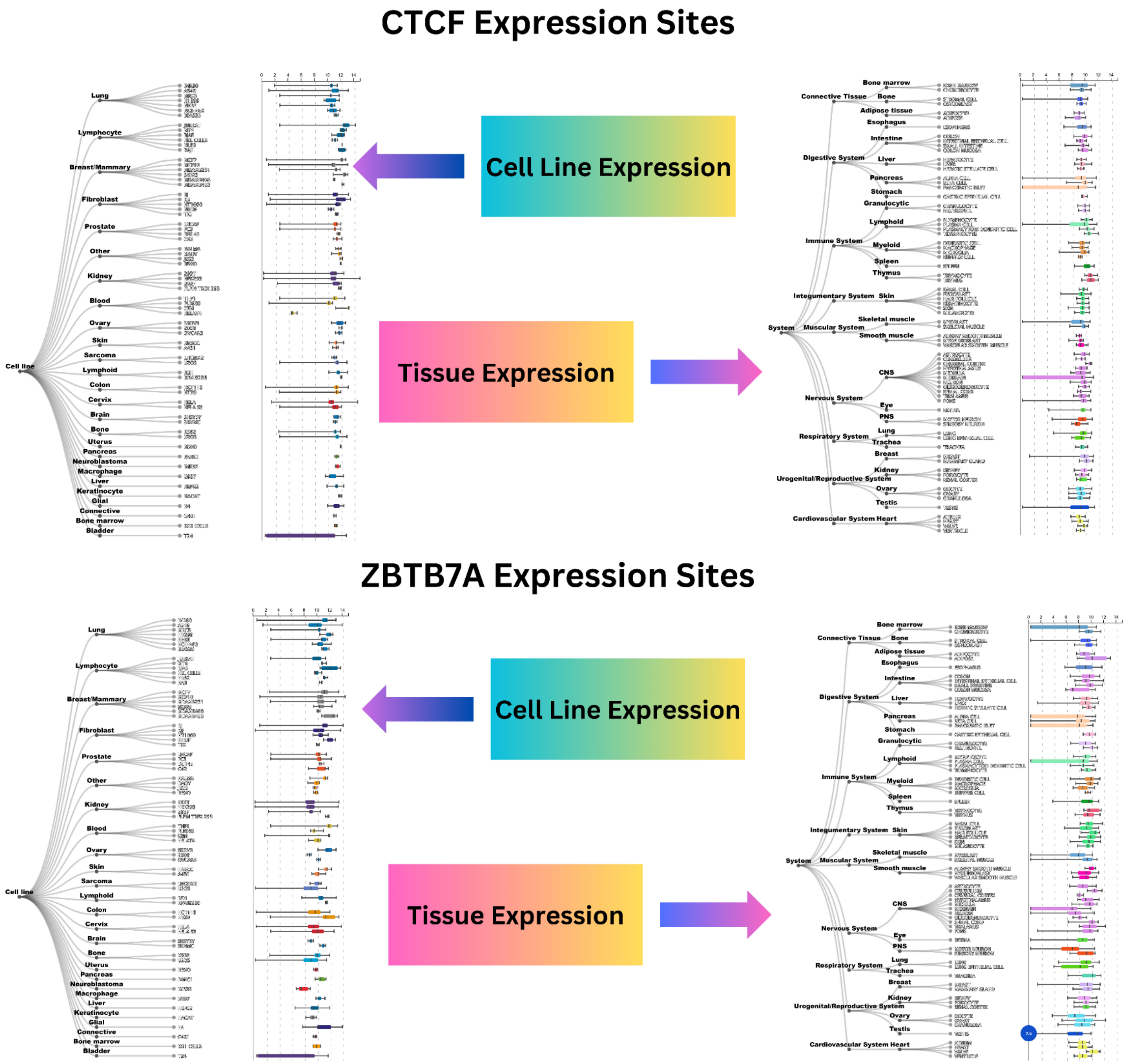

Figure S8 – Tissue-based expression levels and cell line enrichment of the screened TFs (CTCF and ZBTB7A) within the CA10 respectively.

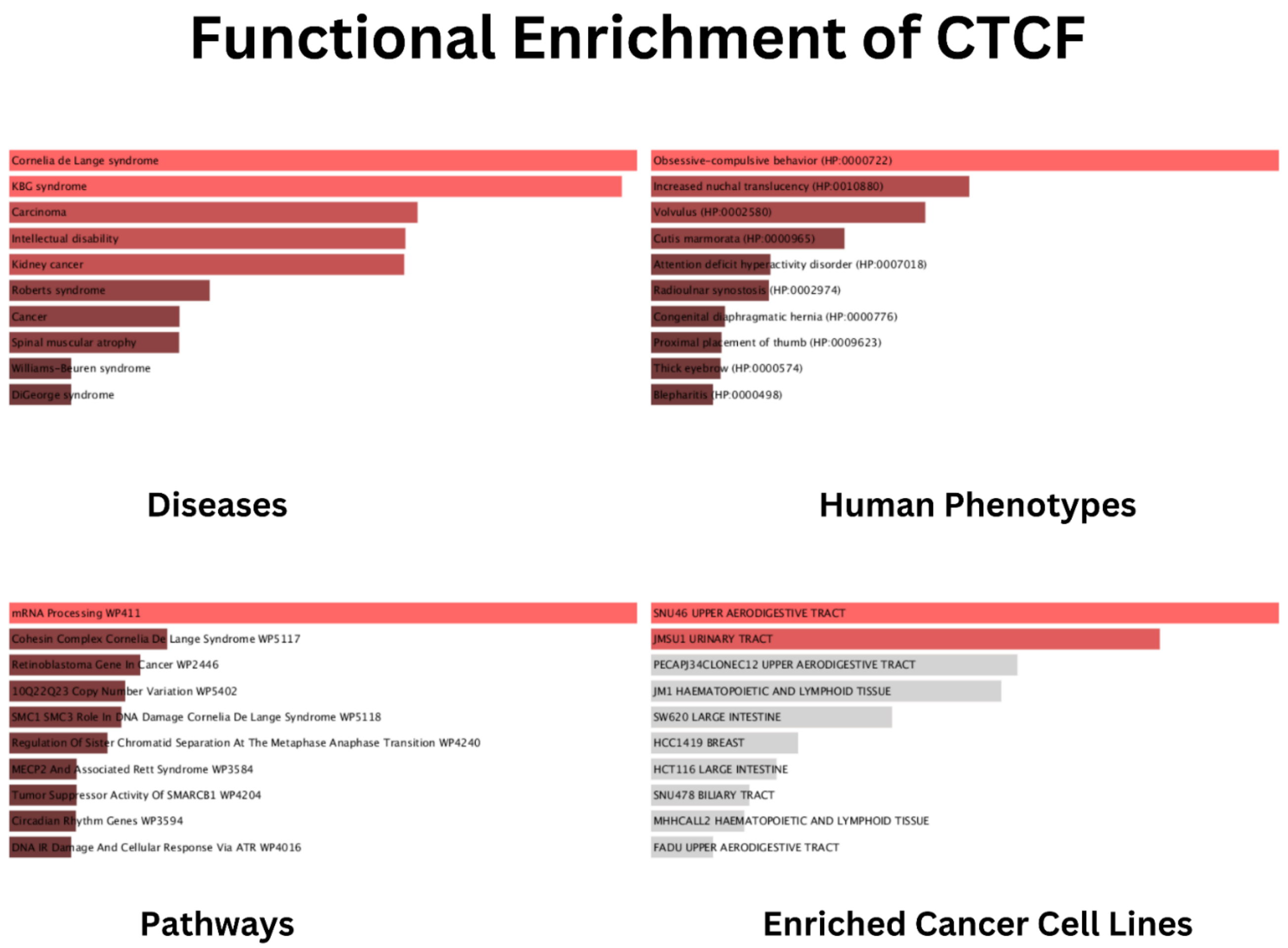

Figure S9 – Functional enrichment analysis of TF: CTCF within CA10

Functional Enrichment of ZBTB7A

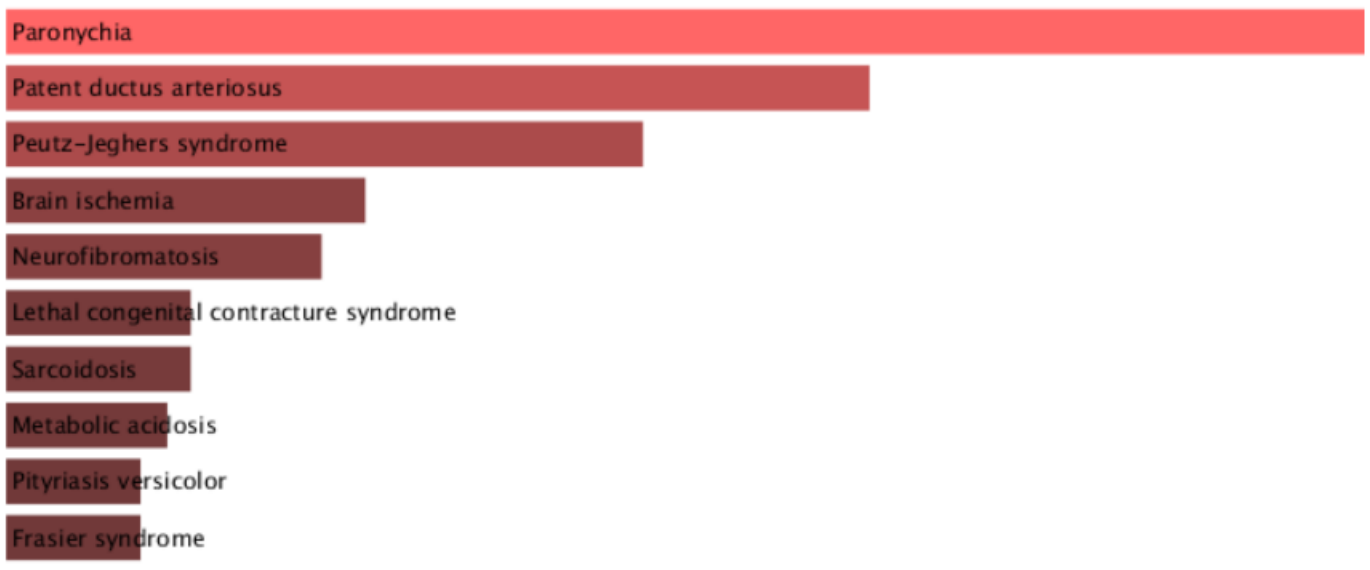

Diseases

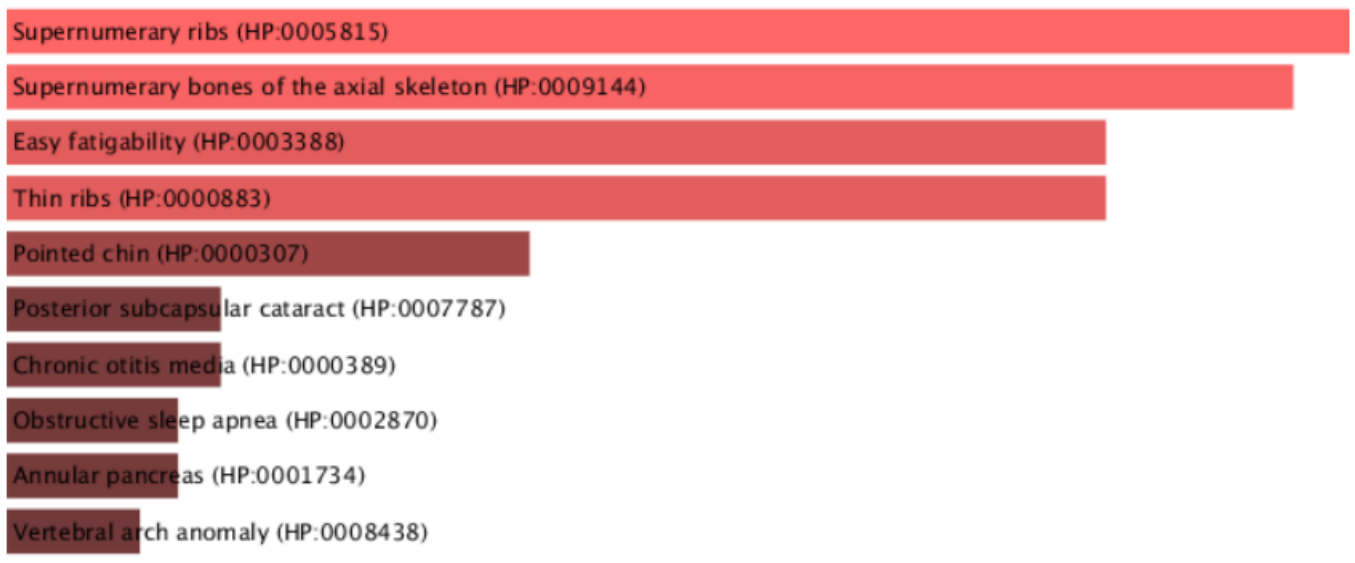

Human Phenotypes

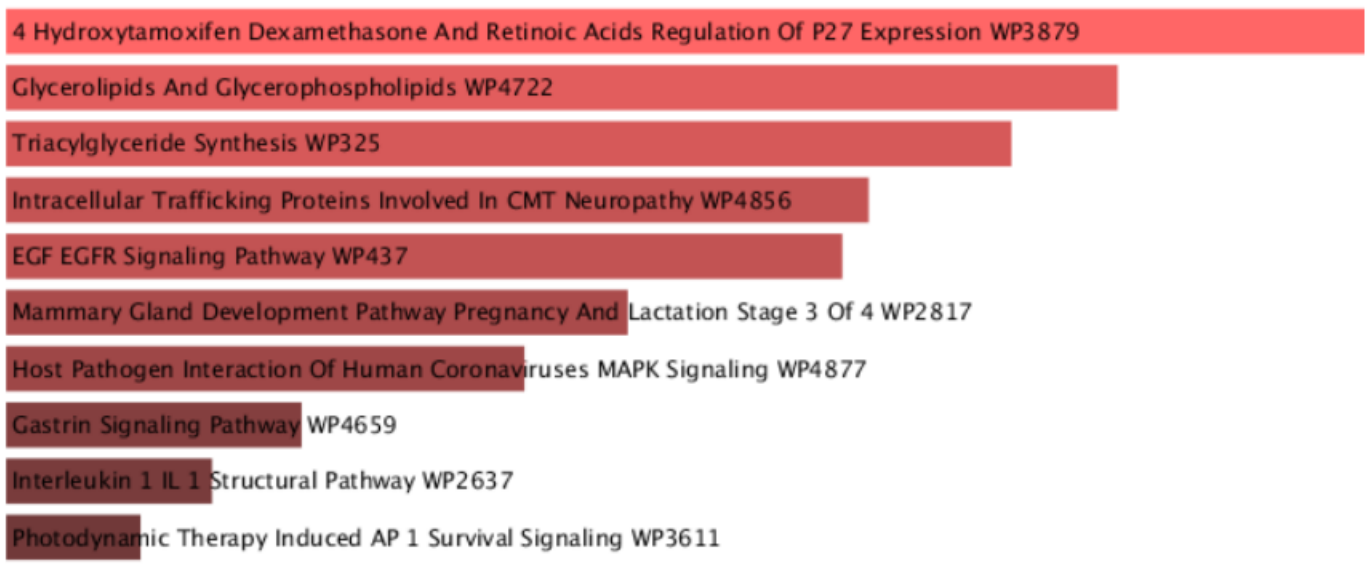

Pathways

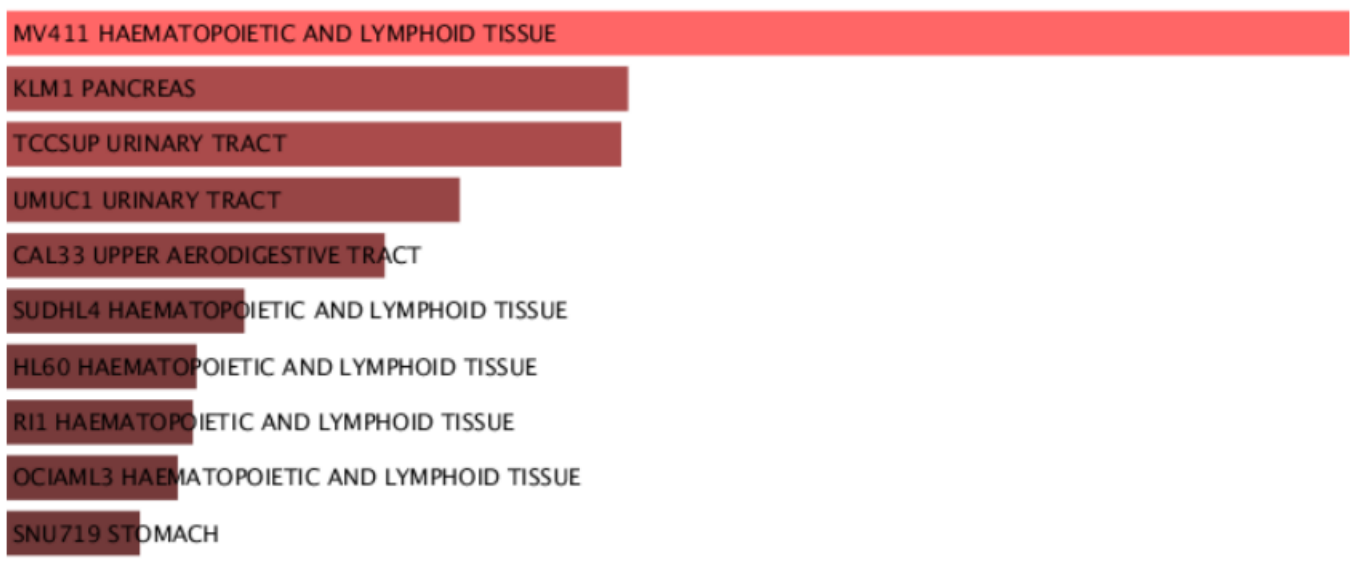

Enriched Cancer Cell Lines

Figure S10 – Functional enrichment analysis of TF: ZBTB7A within CA10

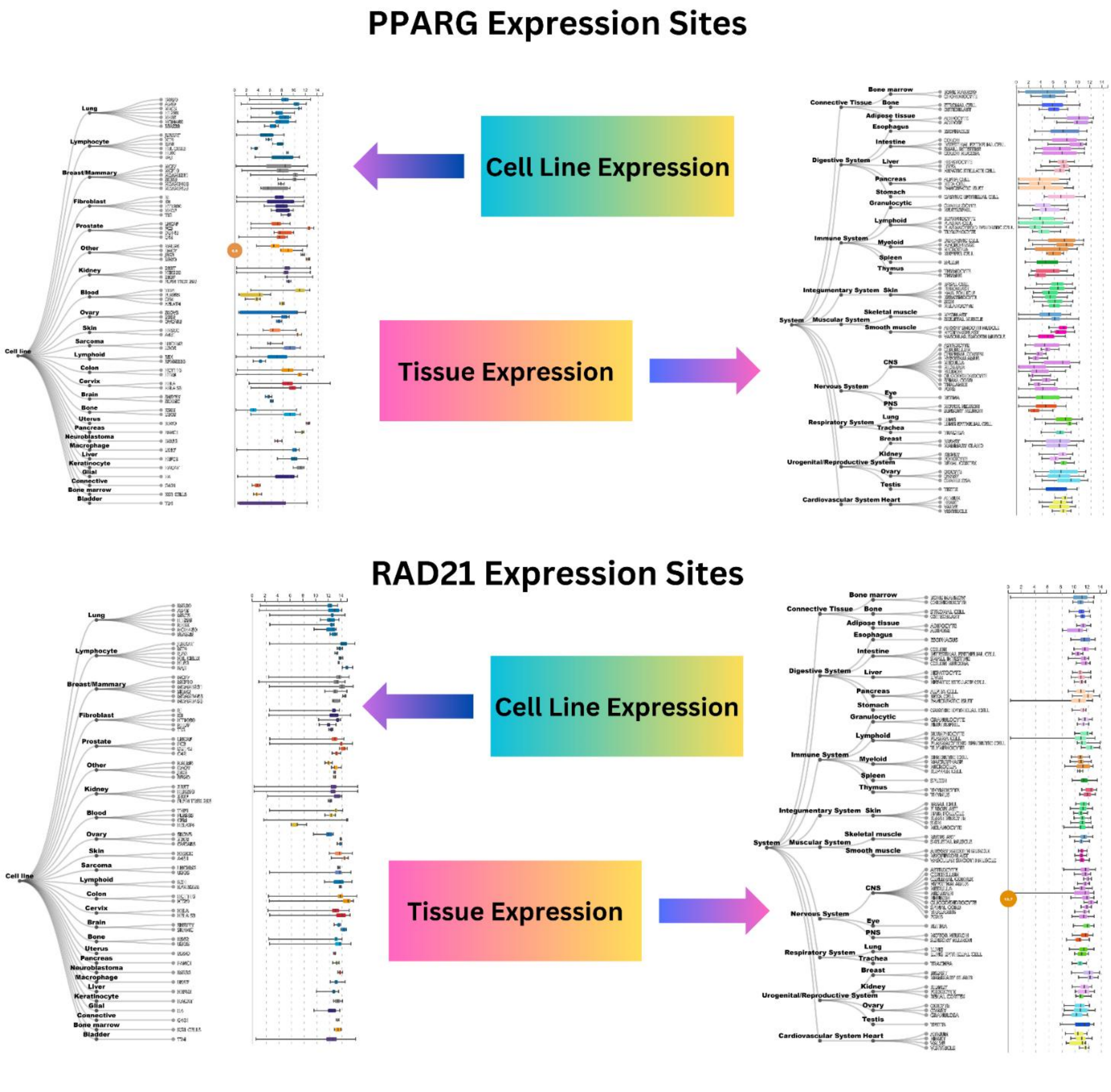

Figure S11 – Tissue-based expression levels and cell line enrichment of the screened TFs (PPARG and RAD21) within the CA11 respectively.

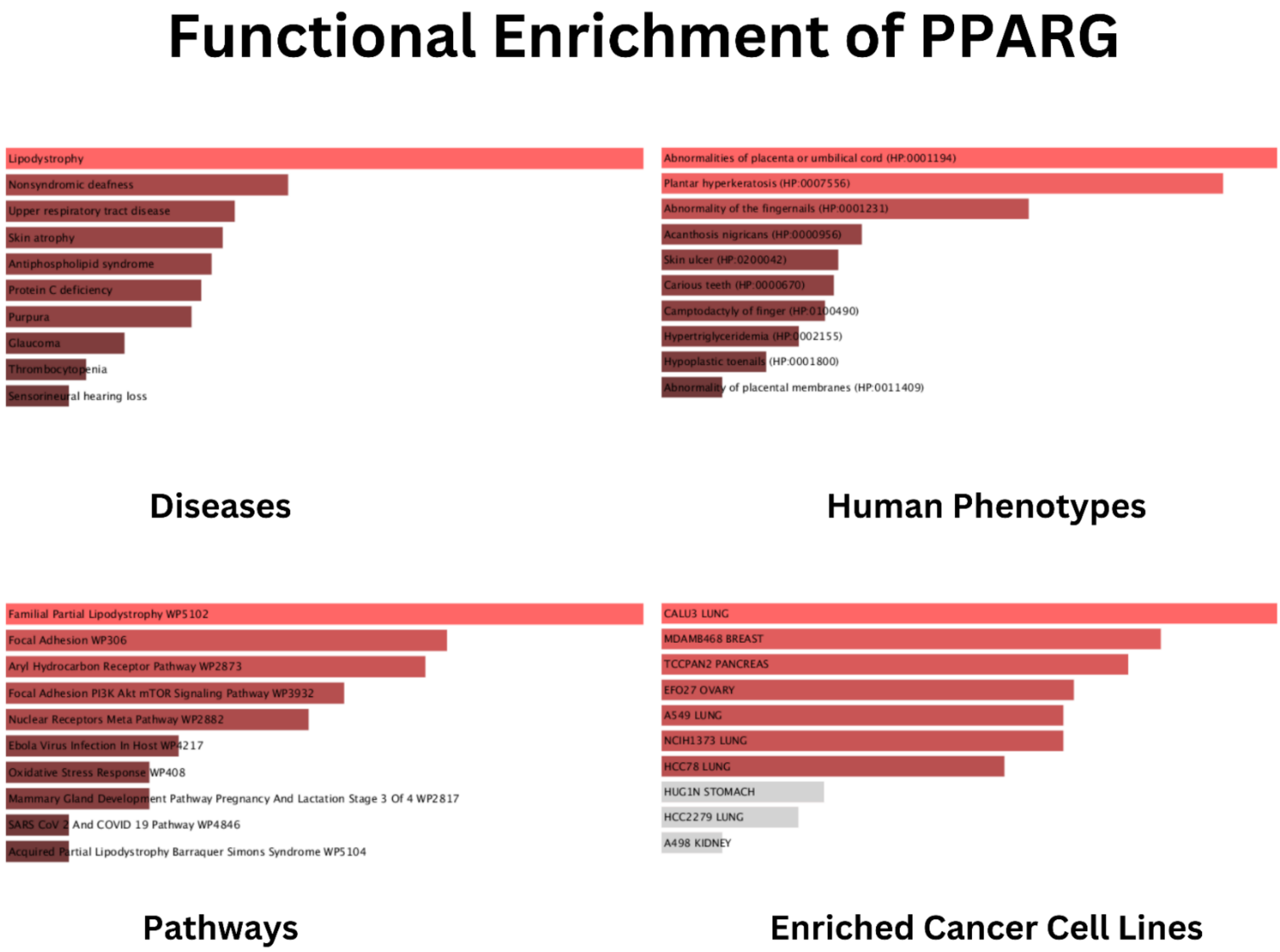

Figure S12 – Functional enrichment analysis of TF: PPARG within CA11

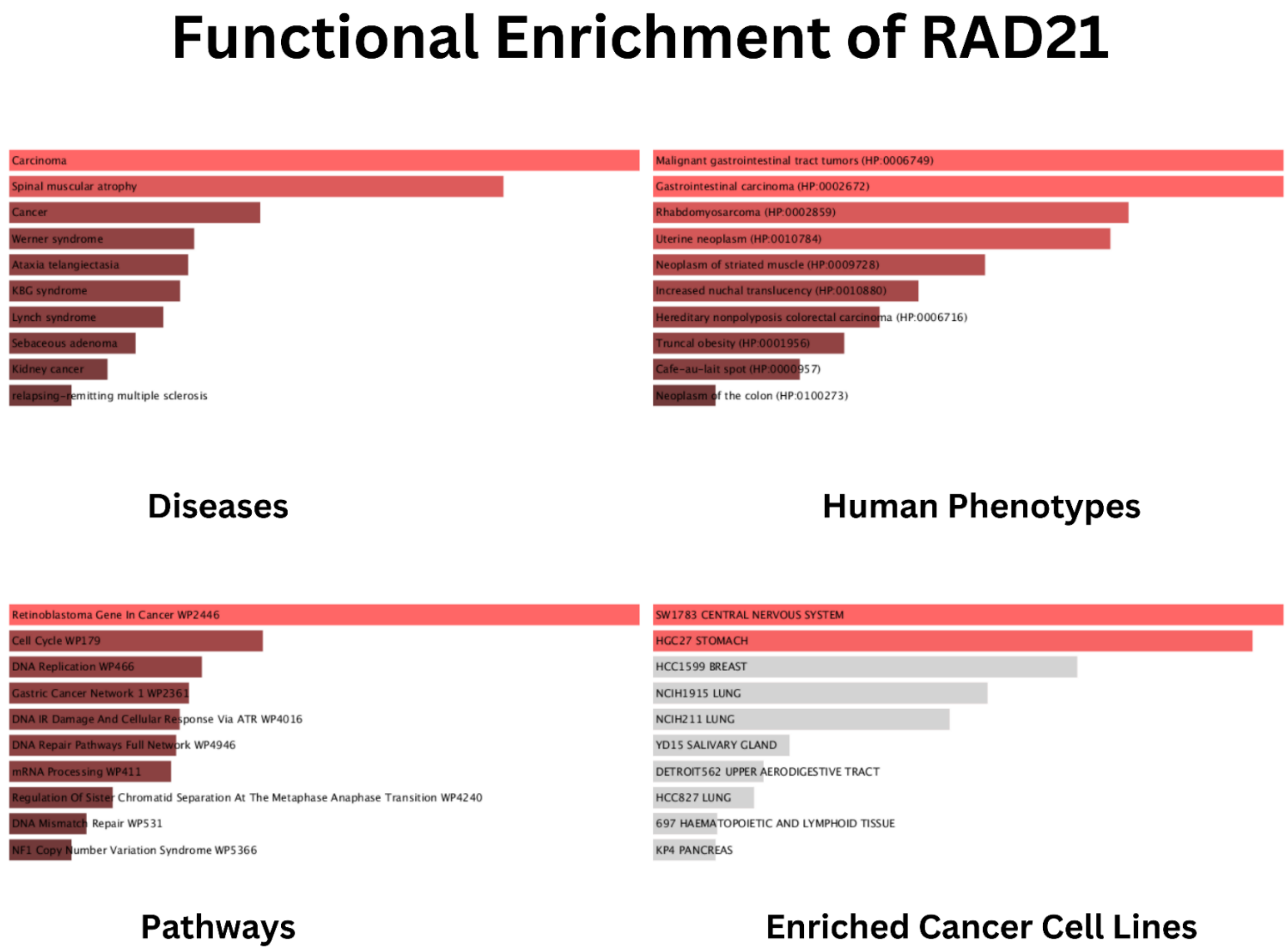

Figure S13 – Functional enrichment analysis of TF: RAD21 within CA11

From the coexpression network of genes associated with CA 8, representation explaining the expression of the respective genes within tissues involved in cancer development based on microarray analysis

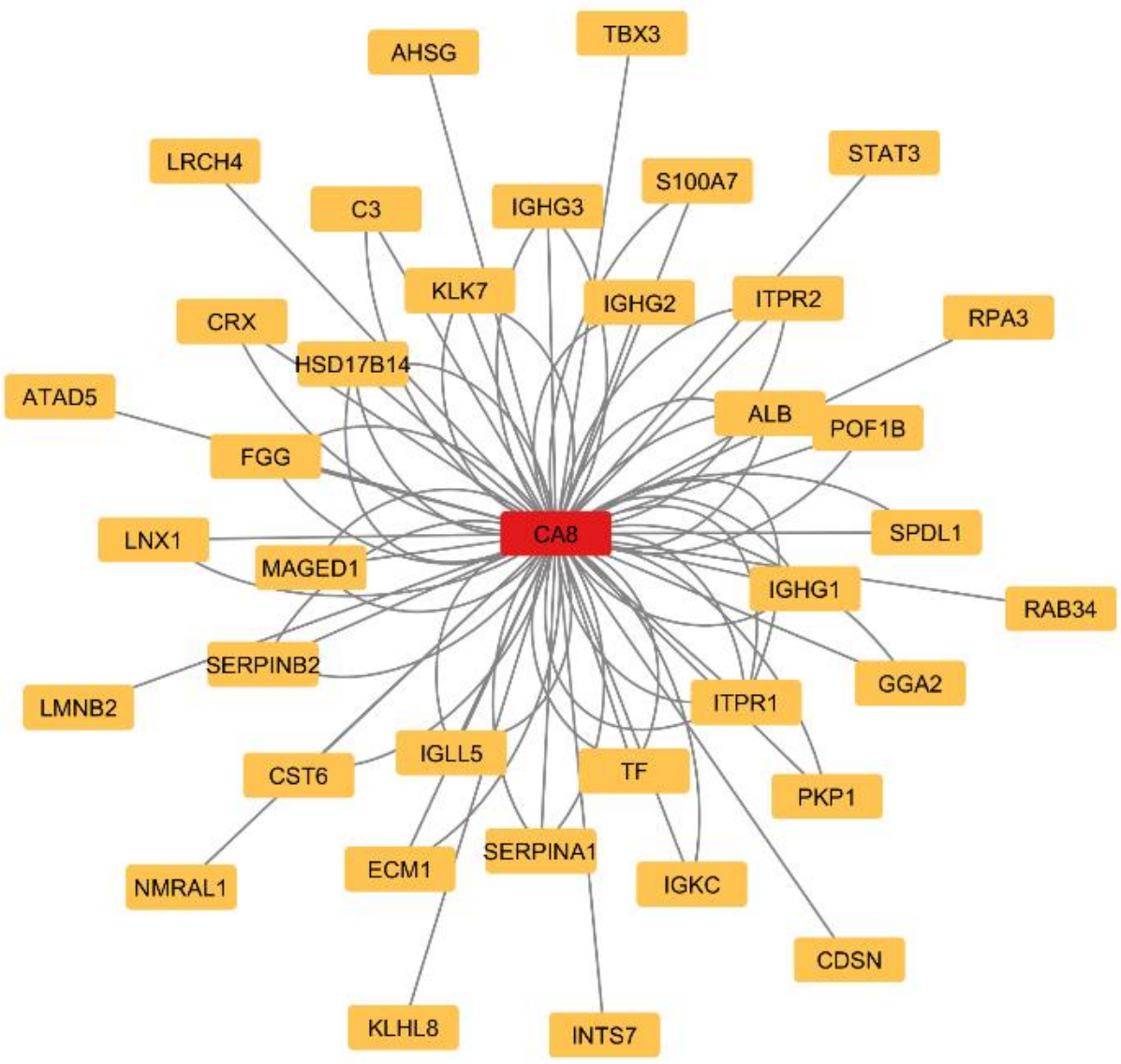

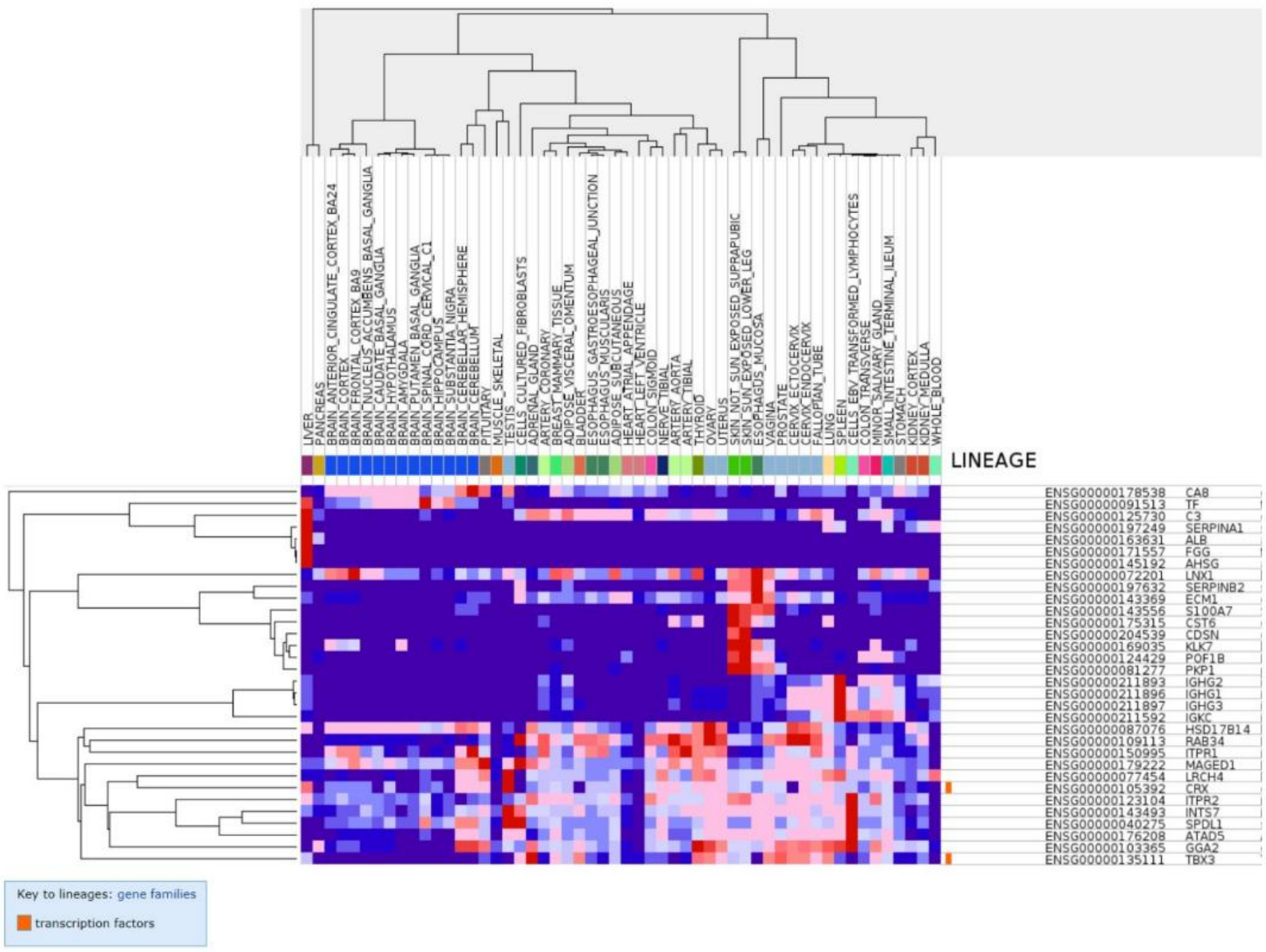

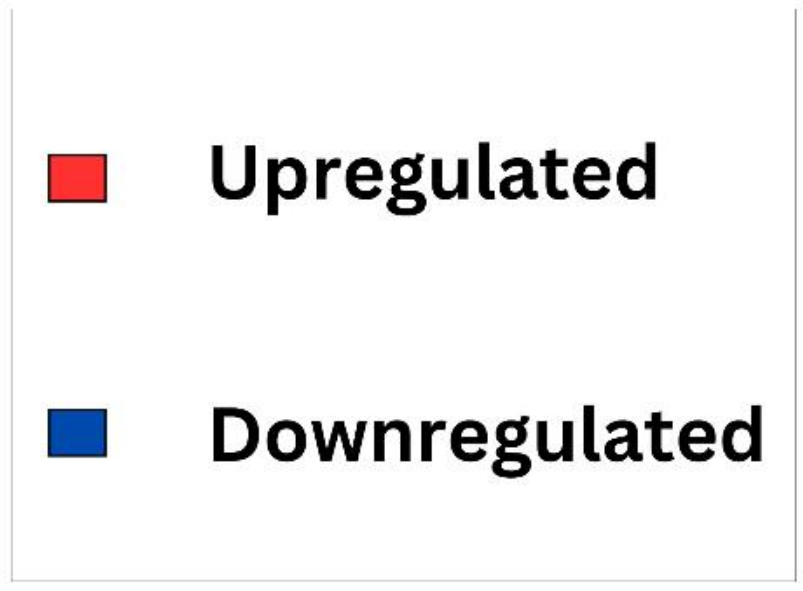

Figure S14 – Coexpression network of genes associated with CA8 and their significant expression levels across multi-organ cancer cell lines through microarray analysis.

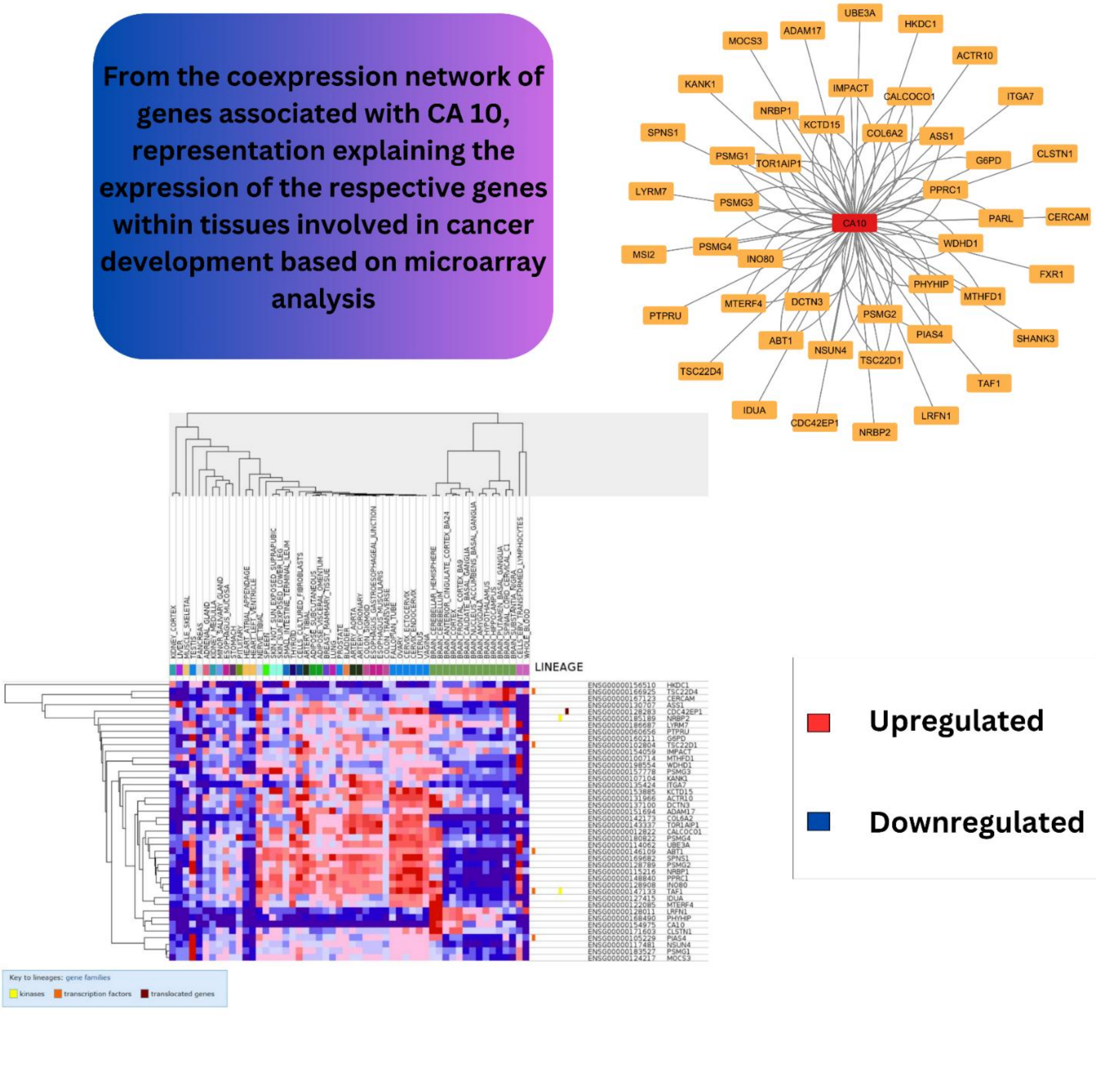

Figure S15 – Coexpression network of genes associated with CA10 and their significant expression levels across multi-organ cancer cell lines through microarray analysis.

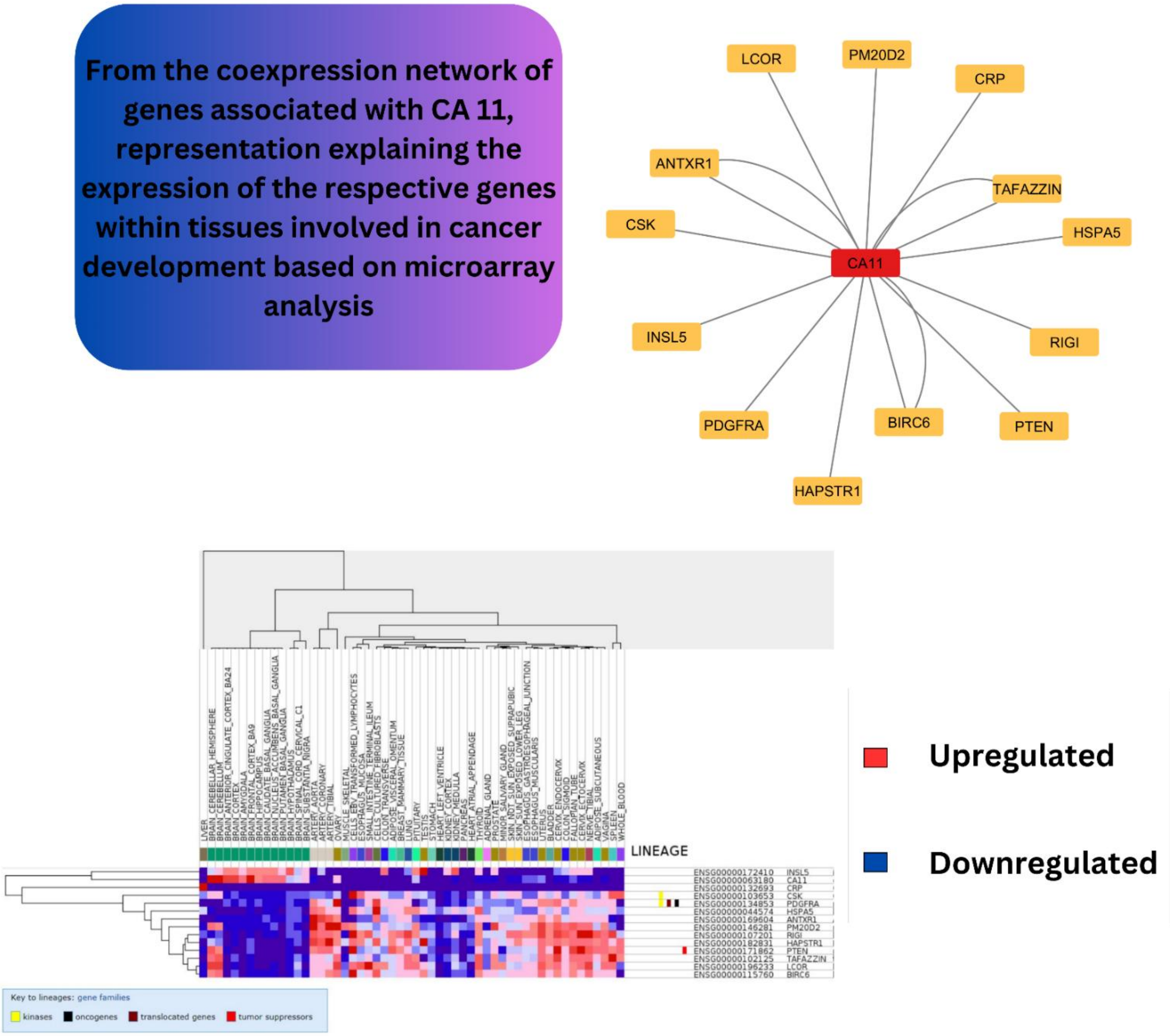

Figure S16 – Coexpression network of genes associated with CA11 and their significant expression levels across multi-organ cancer cell lines through microarray analysis.
